# Biological sex affects the neurobiology of autism

**DOI:** 10.1093/brain/awt216

**Published:** 2013-08-09

**Authors:** Meng-Chuan Lai, Michael V. Lombardo, John Suckling, Amber N. V. Ruigrok, Bhismadev Chakrabarti, Christine Ecker, Sean C. L. Deoni, Michael C. Craig, Declan G. M. Murphy, Edward T. Bullmore, MRC AIMS Consortium, Simon Baron-Cohen

**Affiliations:** 1 Autism Research Centre, Department of Psychiatry, University of Cambridge; Douglas House, 18B, Trumpington Road, Cambridge CB2 8AH, UK; 2 Department of Psychiatry, College of Medicine, National Taiwan University; No.1 Jen-Ai Road Section 1, Taipei 10051, Taiwan; 3 Brain Mapping Unit, Department of Psychiatry, University of Cambridge; Herchel Smith Building, Robinson Way, Cambridge CB2 0SZ, UK; 4 School of Psychology and Clinical Language Sciences, Centre for Integrative Neuroscience and Neurodynamics, University of Reading; Earley Gate, Whiteknights Road, Reading RG6 6AL, UK; 5 Department of Forensic and Neurodevelopmental Sciences, Institute of Psychiatry, King’s College London; PO23, Institute of Psychiatry, De Crespigny Park, Denmark Hill, London SE5 8AF, UK; 6 Advanced Baby Imaging Lab, School of Engineering, Brown University; Wayland Square, 229 Waterman Street, Providence, RI, USA; 7 GlaxoSmithKline, Clinical Unit Cambridge, Addenbrooke’s Hospital, Cambridge CB2 2QQ, UK; 8 Cambridgeshire and Peterborough NHS Foundation Trust, Cambridge, UK

**Keywords:** autism, brain, sex differences, volumetric MRI

## Abstract

In autism, heterogeneity is the rule rather than the exception. One obvious source of heterogeneity is biological sex. Since autism was first recognized, males with autism have disproportionately skewed research. Females with autism have thus been relatively overlooked, and have generally been assumed to have the same underlying neurobiology as males with autism. Growing evidence, however, suggests that this is an oversimplification that risks obscuring the biological base of autism. This study seeks to answer two questions about how autism is modulated by biological sex at the level of the brain: (i) is the neuroanatomy of autism different in males and females? and (ii) does the neuroanatomy of autism fit predictions from the ‘extreme male brain’ theory of autism, in males and/or in females? Neuroanatomical features derived from voxel-based morphometry were compared in a sample of equal-sized high-functioning male and female adults with and without autism (*n = *120, *n = *30/group). The first question was investigated using a 2 × 2 factorial design, and by spatial overlap analyses of the neuroanatomy of autism in males and females. The second question was tested through spatial overlap analyses of specific patterns predicted by the extreme male brain theory. We found that the neuroanatomy of autism differed between adult males and females, evidenced by minimal spatial overlap (not different from that occurred under random condition) in both grey and white matter, and substantially large white matter regions showing significant sex × diagnosis interactions in the 2 × 2 factorial design. These suggest that autism manifests differently by biological sex. Furthermore, atypical brain areas in females with autism substantially and non-randomly (*P* < 0.001) overlapped with areas that were sexually dimorphic in neurotypical controls, in both grey and white matter, suggesting neural ‘masculinization’. This was not seen in males with autism. How differences in neuroanatomy relate to the similarities in cognition between males and females with autism remains to be understood. Future research should stratify by biological sex to reduce heterogeneity and to provide greater insight into the neurobiology of autism.

## Introduction

Autism is a heterogeneous neurodevelopmental condition affecting ∼1% of the general population (Baron-Cohen *et al.*, 2009; Brugha *et al.*, 2011; Mattila *et al.*, 2011; Idring *et al.*, 2012). It is more prevalent in males, with a male:female sex ratio in the range 2:1 to 3:1 (Kim *et al.*, 2011; Mattila *et al.*, 2011; Idring *et al.*, 2012). Most biological studies of autism have predominantly focused on males, which may have potentially resulted in a male-biased view of the neurobiology of autism. For example, the male-bias in research samples is ∼8:1 in neuroimaging studies of brain volume (Via *et al.*, 2011), and 15:1 in task-functional MRI studies (Philip *et al.*, 2012). Autism in females has not attracted the same level of attention and has been assumed to be similar to that in males. Biological sex may contribute significantly to the heterogeneity in autism, and ignoring potential sex differences within autism may underlie non-replication of research results. For instance, re-analysis of genome-wide association study data when modelling in sex-specific effects illuminates new genetic markers that were not detected when sex-specificity is ignored (Lu and Cantor, 2012). Separating males and females may thus be a useful way forward for uncovering important risk and protective mechanisms in the development of autism (Werling and Geschwind, 2013). In this study we ask two fundamental questions to help understand how biological sex affects the neurobiology of autism:

(i) Is the neuroanatomy of autism different in males and females? Growing evidence suggests that females with autism differ from males at multiple levels. Behaviourally females may go undetected due to a ‘non-male-typical’ presentation or a greater ability to camouflage their difficulties (Attwood, 2006; Baron-Cohen *et al.*, 2011; Kopp and Gillberg, 2011). Although some studies report no sex differences in cardinal autistic behavioural characteristics after controlling for IQ (Tsai and Beisler, 1983; Pilowsky *et al.*, 1998; Holtmann *et al.*, 2007; Solomon *et al.*, 2012), others do (McLennan *et al.*, 1993; Carter *et al.*, 2007; Hartley and Sikora, 2009; Lai *et al.*, 2011; Mandy *et al.*, 2012). Females with autism have also been found to differ from males with autism at the levels of cognition (Carter *et al.*, 2007; Bolte *et al.*, 2011; Lemon *et al.*, 2011; Lai *et al.*, 2012*b*), proteomics (Schwarz *et al.*, 2011; Ramsey *et al.*, 2012), hormones (Ruta *et al.*, 2011; Bejerot *et al.*, 2012), genetics (Gilman *et al.*, 2011; Puleo *et al.*, 2012; Szatmari *et al.*, 2012), transcriptomics (Kong *et al.*, 2012), and early brain overgrowth (Sparks *et al.*, 2002; Bloss and Courchesne, 2007; Schumann *et al.*, 2009, 2010; Nordahl *et al.*, 2011). Structural neuroimaging studies focusing on females (Craig *et al.*, 2007; Calderoni *et al.*, 2012) also reveal little overlap of atypical brain areas compared with those found in meta-analyses of predominantly male samples (Radua *et al.*, 2011; Via *et al.*, 2011). In short, though defined by the same diagnostic criteria (American Psychiatric Association, 2000), autism in males and females may involve different biological underpinnings.
(ii) Does the neuroanatomy of autism fit predictions from the ‘extreme male brain’ (EMB) theory of autism, in males and/or in females? The EMB theory proposes that autism represents an amplification of specific aspects of typical sexual dimorphism in cognition (e.g. empathy and systemizing) (Asperger, 1944, 1991; Wing, 1981; Baron-Cohen, 2002). Specific biological mechanisms that influence the expression of sexual dimorphism are thought to underlie this ‘masculinization’ (Baron-Cohen *et al.*, 2011). In view of the sex differences within autism illustrated earlier, the most appropriate way to test this theory is to investigate males and females separately. At physiological and behavioural levels, previous observations in females seem to particularly fit predictions from the EMB theory. Compared to typically developing females, girls with autism show decreased female-typical play (Knickmeyer *et al.*, 2008) and behaviours (Ingudomnukul *et al.*, 2007), and women with autism have a higher rate of androgen-related medical/developmental conditions such as polycystic ovary syndrome (Ingudomnukul *et al.*, 2007) and late onset of menarche (Knickmeyer *et al.*, 2006), and showed elevated serum testosterone level and masculinized physical features (Schwarz *et al.*, 2011; Bejerot *et al.*, 2012). Females but not males with autism also show an atypical serum proteomic profile that includes androgen-related molecules (Schwarz *et al.*, 2011). Both males and females with autism have elevated serum levels of androstenedione, the precursor to testosterone, but the effect size is larger in females (Ruta *et al.*, 2011). Together this suggests that in females with autism, atypical androgen-related mechanisms, if aetiologically related, may be more evident than in males with autism.

In the present study we test, to our knowledge, the largest sample to date of high-functioning male and female adults with autism, with the aim of answering these two questions by comparing neuroanatomy measured in terms of voxel-based morphometry (VBM) (Ashburner and Friston, 2000), a well-established method for observing local volumetric differences in an unbiased whole-brain mass-univariate statistical framework. An unbiased whole-brain approach provides a better overview than focusing on limited numbers of regions of interest in answering these research questions, in light of the substantial heterogeneity in the neurobiology of autism and the limited understanding to that in females to date.

## Materials and methods

### Participants

Participants (*n = *120) included 30 right-handed pre-menopausal females and 30 males with autism, along with 30 neurotypical females and 30 neurotypical males. All groups were matched for age (18–49 years) and full-scale IQ. Participants with autism had a formal clinical diagnosis of International Classification of Diseases-10 (World Health Organization, 1992) childhood autism or Asperger’s syndrome, or Diagnostic and Statistical Manual of Mental Disorders-IV text revision (American Psychiatric Association, 2000) autistic disorder or Asperger’s disorder assessed by a psychiatrist or clinical psychologist in the National Health Service, UK. They all reached the diagnostic algorithm cut-offs on the Autism Diagnostic Interview-Revised (Lord *et al.*, 1994) (with the exception of two females for whom Autism Diagnostic Interview-Revised data were unavailable). One point below in only one of the three core symptom domains was permitted, to allow for possible underestimation of early developmentally atypical behaviours in the recall of caregivers whose children were now adults. Autism Diagnostic Observation Schedule (Lord *et al.*, 2000) module 4 was performed but the score was not used as an inclusion criterion due to its potentially unsatisfactory sensitivity to high-functioning adults with autism, particularly females (Lai *et al.*, 2011). These all followed our earlier studies and rationale for inclusion (Lai *et al.*, 2010, 2011, 2012*b*; Lombardo *et al.*, 2010, 2011; Ecker *et al.*, 2012, 2013). For the two females without available Autism Diagnostic Interview-Revised information (their childhood caregivers could not be interviewed), one scored above the cut-off for ‘autism spectrum’ on the Autism Diagnostic Observation Schedule and the other was positive for a diagnosis on the Adult Asperger Assessment, which incorporates caregiver reports of childhood behaviours and developmental history (Baron-Cohen *et al.*, 2005). Exclusion criteria for all groups included history of or current psychotic disorders, substance-use disorders, severe head injury, genetic disorders associated with autism (e.g. fragile × syndrome, tuberous sclerosis), intellectual disability (i.e. IQ <70), hyperkinetic disorder, Tourette’s disorder or any other medical condition significantly affecting brain function (e.g. epilepsy). The neurotypical groups did not have autism either themselves or in their family history.

All participants were recruited through the UK Medical Research Council Autism Imaging Multicentre Study (MRC AIMS) and were assessed at the Autism Research Centre, University of Cambridge. Informed written consent was obtained for all participants in accord with procedures approved by the Suffolk Research Ethics Committee. Further recruitment details can be found elsewhere (Lai *et al.*, 2011, 2012*b*; Ecker *et al.*, 2012, 2013).

All participants were assessed by the Wechsler Abbreviated Scale of Intelligence (Wechsler, 1999), questionnaires measuring autistic traits (Autism Spectrum Quotient; Baron-Cohen *et al.*, 2001*b*); empathy (Empathy Quotient; Baron-Cohen and Wheelwright, 2004) and self-awareness of own emotions (Toronto Alexithymia Scale; Bagby *et al.*, 1994), and an advanced mentalizing task (‘Reading the Mind in the Eyes’ test; Baron-Cohen *et al.*, 2001*a*). Participants’ second and fourth digit lengths (2D:4D ratio) of both hands were measured as a proxy of prenatal hormonal influence (Breedlove, 2010), using an electronic vernier caliper (Supplementary material).

### Structural magnetic resonance imaging acquisition and preprocessing

All 120 participants were scanned using a contemporary 3 T MRI scanner (GE Medical Systems HDx) fitted with an 8-channel receive-only RT head-coil. A specialized acquisition protocol employing quantitative imaging (Driven Equilibrium Single Pulse Observation of T_1_, DESPOT1; see Supplementary material) was used (Deoni *et al.*, 2008), which has been applied in large-scale multicentre studies (Ecker *et al.*, 2012, 2013; Lai *et al.*, 2012*a*). Simulated T_1_-weighted inversion recovery images derived from DESPOT1 were segmented and normalized to the standard Montreal Neurological Institute (MNI) space using the SPM8 software (Wellcome Trust Centre for Neuroimaging, London, UK). Native space grey matter, white matter and CSF images were obtained using standard automated segmentation routines. Individual total grey matter, white matter and CSF volumes were estimated by summing up the partial volume estimates throughout each class of image in the native space. The native space grey and white matter images were registered to a study-specific template using a high-dimensional non-linear diffeomorphic registration algorithm (DARTEL) (Ashburner, 2007). A modulation step was included to retain voxel-wise information about local tissue volume. The modulated grey and white matter maps were smoothed with a 4 mm full-width at half-maximum Gaussian kernel.

### Statistical analytic strategies for the two research questions

Voxel-wise statistical tests (i.e. VBM) were performed with SPM8. To avoid possible edge effects between different tissue types, the grey matter group comparisons were constrained within the grey matter segment of the study-specific template image with a threshold of partial volume estimates > 0.25. A parallel procedure was introduced for the white matter group comparisons. Before statistical modelling, each modulated grey/white matter map was rescaled by individual total grey/white matter volume (i.e. voxel value divided by individual total volume) to derive a map indicating relative grey/white matter volume. Individual-level rescaling was performed in a tissue-specific manner (rather than using total brain volume) for the reason that the relationship between grey and white matter volumes is not linear (Zhang and Sejnowski, 2000), so correction by total brain volume would be less appropriate for our purpose of observing tissue-specific local variations.

Using the whole-brain data, the research questions were addressed at two levels conjointly: first in a (mass)univariate sense to investigate the pattern of magnitude differences across groups; second in a multivariate sense to establish the pattern of spatial distribution in the brain of the group-differences.

#### Analytic strategy to Question 1: Is the neuroanatomy of autism different in males and females?

At the magnitude level, the presence of a significant sex × diagnosis interaction in a 2 × 2 factorial design suggests that atypical neuroanatomical features of autism manifest differently as a function of biological sex. Here we fit a general linear model at each voxel, with sex and diagnosis as fixed factors and age a nuisance covariate, to test for significant interactions. At the spatial distribution level, the presence of significantly large contiguous clusters, rather than isolated small clusters of voxels, indicates that substantial brain regions show statistical significance in the tests. Therefore for VBM (for both grey and white matter), statistical outcomes were corrected for multiple comparisons at the cluster level by controlling topological false discovery rate (FDR) calculated under Gaussian Random Field Theory (Chumbley and Friston, 2009), using a cluster-forming voxel-level height threshold of *P < *0.025 for each contrast and a spatial extent threshold (corrected for non-stationarity) Hayasaka *et al.*, 2004) that ensures a cluster-wise FDR at *q* < 0.05. Labelling of white matter anatomical structures was done by overlaying the significant clusters with standard-space white matter tracts probabilistically defined from a human diffusion tensor imaging atlas (Thiebaut de Schotten *et al.*, 2011).

#### Analytic strategy to Question 2: Does the neuroanatomy of autism fit predictions from the EMB theory of autism, in males and/or in females?

At the magnitude (univariate) level, the EMB theory suggests that autism coincides with an amplification of typical sexual dimorphism. What is key is the matching of directionality between two group-difference patterns (e.g. the effects of autism and sex on scores of an empathy task act in the same direction by the following pattern: males < females AND autism < neurotypical controls). Therefore, EMB theory predictions are confirmed only if the following prerequisite M1 is established AND requisite M2 and/or M3 is true:

Prerequisite M1: There is a statistically significant sexual dimorphism in the typically developing population [i.e. for a measure, neurotypical male control subjects (MC) > neurotypical female control subjects (FC), or vice versa].
Requisite M2: Males with autism (MA) are more ‘masculinized’ compared with neurotypical male control subjects (MC) (i.e. MA > MC, or vice versa).
Requisite M3: Females with autism (FA), though not explicitly described in the original formulation, should perform similarly to males with autism, thus are more ‘masculinized’ compared to neurotypical female control subjects (i.e. FA > FC, or vice versa).

Given these, EMB theory predictions in the brain (which is in the spatial domain and multivariate in nature) should be tested by spatial overlap analyses on three planned VBM between-group comparisons (MC–FC, MA–MC, FA–FC), which themselves have shown magnitude-level effects (Fig. 2A). By the same rationale, the prerequisite and requisites will be:

Prerequisite S1: There is a typical sexual dimorphism in the brain (e.g. for volume, MC > FC in region X).
Requisite S2: The group-difference map between males with autism and male control subjects matches in the directionality predicted by the EMB theory with, and spatially overlaps substantially with, the group-difference map between male and female control subjects (e.g. MA > MC in region Y, and Y overlaps with X).
Requisite S3: The group-difference map between females with autism and female control subjects matches in the directionality predicted by the EMB theory with, and spatially overlaps substantially with, the group-difference map between male and female conrol subjects (e.g. FA > FC in region Z, and Z overlaps with X).

If both Prerequisite S1 and Requisite S2 are true, the EMB theory prediction in males is confirmed; if both Prerequisite S1 and Requisite S3 are true, the prediction in females is confirmed; if all Prerequisite S1, Requisites S2 and S3 are true, the predictions for both males and females are confirmed. In this last instance, one will also expect to see substantial spatial overlap between the main effect maps from the earlier 2 × 2 factorial design VBM (i.e. male > female overlaps with autism > control, and female > male overlaps with control > autism).

Three sets of planned VBM comparisons (MC–FC, MA–MC, FA–FC; Fig. 2A) on relative grey and white matter volumes were first performed, with two contrasts in each (e.g. for MC–FC, there were MC > FC and FC > MC). For spatial overlap analyses, we applied only voxel-level height thresholds and no spatial extent thresholds. This is because using a topological FDR procedure to control for type I error will result in different spatial extent thresholds for different VBM comparisons, potentially influencing the overlap analyses across group-difference maps. We did not apply a common (arbitrary) extent threshold (e.g. 100 voxels) as we were also examining how overlapping voxels were spatially distributed (i.e. contiguous versus dispersed). The extent of overlap was measured along maps thresholded from voxel-level *P < *0.05 down to *P < *0.0001 to illustrate if the pattern was consistent and stable.

For each set of spatial overlap analysis, we performed a conjunction analysis consisting of logical AND masking (Nichols *et al.*, 2005), then computed the overlap as a proportion of the total number of suprathreshold voxels for each map. Each conjunction analysis was performed on the two contrasts following the directionality predicted by the EMB theory (testing Requisite S2: MA > MC AND MC > FC, MC > MA AND FC > MC; testing Requisite S3: FA > FC AND MC > FC, FC > FA AND FC > MC). To test for statistical significance, we ran Monte Carlo simulations (5000 iterations) to create the null distribution of random overlaps at each voxel-level threshold from *P = *0.05 to *P = *0.0001 (500 in total, black lines in Fig. 2, and Supplementary Fig. 3) to assess the probability that the overlap did not occur by random (Lombardo *et al.*, 2012*b*).

For each iteration of the Monte Carlo simulation we generated two whole-grey matter/white matter maps filled with values sampled randomly from a Gaussian distribution and having the same smoothness as the observed group-difference maps. These simulated maps were then thresholded at the same voxel-level threshold as the observed maps, and the percentage of overlapping voxels in the two suprathreshold simulated maps was calculated. Over the 5000 iterations we constructed the null distribution of the overlap percentage that occurred by random. *P*-values from this simulation were computed by counting the number of instances where overlapping percentages were greater than or equal to the observed overlapping percentage in the real data. A low *P*-value (e.g. < 0.001) indicates that the observed overlap does not occur by chance; a high *P-*value (e.g. > 0.999) indicates that the observed overlap represents a significant non-overlap and/or is generated from non-random maps. All computations were performed with MATLAB version 2008a (The MathWorks Inc., Natick, MA, USA).

##### Additional spatial overlap analysis using a larger multicentre male sample

Unlike the MC–FC and FA–FC comparisons, group-differences between males with and without autism in the main sample (*n = *30/group) were relatively sparse and of small effect sizes. Therefore an additional MA–MC VBM was conducted on a larger multicentre male sample from the MRC AIMS project (Ecker *et al.*, 2013) to provide greater power to detect the diagnostic group differences within males. Simulated T_1_-weighted inversion-recovery images derived from DESPOT1 composed of 84 neurotypical adult males and 84 males with autism matched for age and full-scale IQ were compared by VBM (Supplementary material). All preprocessing steps and statistical inference procedures were done in the same way as described earlier for the main sample, except (i) the DARTEL template-creation and normalization included only these 168 male participants; and (ii) in the general linear model for VBM, centres (i.e. scanning machines) were also included as covariates (categorical fixed-effect factors).

#### Correlation with 2D:4D ratio

Pearson’s correlation was used to demonstrate the relationship between relative volume of the overlapping regions to 2D:4D ratio in the female groups. By constructing a linear regression model with volume as the dependent variable and group, 2D:4D ratio and group × 2D:4D ratio as regressors, significance of group difference on the correlations was assessed by the *β* (and *P*-value) for the interaction term ‘group × 2D:4D ratio’. These analyses were performed with the PASW Statistics version 18 (SPSS Inc.).

## Results

### Question 1: Is the neuroanatomy of autism different in males and females?

Age and IQ-matched adult males and females with autism (*n = *30/group) had comparable levels of childhood autistic symptoms, current mentalizing ability and related dispositional traits (Table 1, Supplementary material and Supplementary Fig. 1). Females, however, showed fewer behavioural autistic features during interpersonal interaction but slightly higher self-report autistic traits. This may reflect greater effort at camouflage, greater self-awareness, developmental differences, and/or measurement issues (Lai *et al.*, 2011).

**Table 1:** Participant characteristics

Mean (SD) [range]^a^	Male controls	Males with autism	Female controls	Females with autism	Statistics^b^
Age, years	28.2 (5.6)	27.2 (7.3)	27.5 (6.5)	27.8 (7.6)	ns
Verbal IQ	112.7 (9.7)	114.3 (12.9)	118.5 (9.6)	115.8 (13.1)	ns
Performance IQ^c^	118.5 (11.6)	113.3 (15.0)	117.0 (9.3)	110.4 (16.7)	MC > FA (*P = *0.021)
Full-scale IQ^c^	117.5 (10.7)	115.4 (14.1)	120.2 (8.0)	114.9 (13.8)	ns
Autism Diagnostic Interview-Revised^d^					
Social	–	18.0 (5.1) [10–27]	–	16.4 (4.3) [11–26]	ns
Communication	–	15.3 (3.5) [8–22]	–	13.1 (3.9) [8–22]	MA > FA (*P = *0.029)
Repetitive, restrictive and stereotyped behaviour^c^	–	5.6 (2.5) [2–10]	–	4.3 (1.7) [2–8]	MA > FA (*P = *0.023)
Autism Diagnostic Observation Schedule					
Social interaction + communication total score	–	8.5 (5.0) [1–17]	–	4.3 (3.6) [0–13]	MA > FA (*P < *0.001)
Repetitive, restrictive and stereotyped behaviour	–	1.0 (1.0) [0–4]	–	0.1 (0.3) [0–1]	MA > FA (*P < *0.001)
Autism Spectrum Quotient	15.6 (6.9)	32.7 (7.3)	12.0 (4.8)	37.5 (6.7)	^e^
Empathy Quotient	42.7 (11.9)	19.7 (10.1)	53.5 (9.5)	19.5 (7.5)	^e^
TAS-20	42.5 (10.3)	61.4 (9.2)	41.1 (9.2)	65.5 (8.1)	^e^
Eyes Test	27.0 (3.4)	22.8 (5.8)	28.8 (2.3)	23.4 (6.2)	^e^
Grey matter, cm^3^	914 (78)	940 (105)	824 (81)	845 (72)	^f^
White matter, cm^3^	510 (38)	513 (56)	448 (53)	465 (47)	^f^
CSF, cm^3^	270 (59)	264 (63)	236 (53)	227 (45)	^f^
Total brain volume, cm^3^	1424 (103)	1453 (154)	1272 (124)	1310 (107)	
Total intracranial volume, cm^3^	1695 (135)	1717 (177)	1508 (151)	1537 (118)	

*n* = 30 per group. ns = non-significant (*P* > 0.05).

TAS-20 = Toronto Alexithymia Scale; Eyes Test = Reading the Mind in the Eyes Test.

Total intracranial volume = total brain volume + CSF; total brain volume = grey matter + white matter.

^a^For Autism Diagnostic Interview-Revised and Autism Diagnostic Observation Schedule scores.

^b^Independent sample *t*-tests between any two groups, except non-parametric Mann-Whitney tests for Autism Diagnostic Observation Schedule algorithm scores (distribution significantly deviant from normal). All *P*-values were not corrected for multiple comparisons.

^c^Levene’s Test for Equality of Variances showed significant non-equal variances, therefore equal variance was not assumed.

^d^*n* = 30 for males with autism, *n* = 28 for females with autism.

^e^See Supplementary material and Supplementary Fig. 1 for statistical details of group differences.

^f^See ‘Results’ section for statistical details of group differences.

Globally, a two-way multivariate ANOVA with absolute (i.e. not adjusted by body size) total grey matter, white matter and CSF volumes as dependent variables and sex and diagnosis as fixed factors revealed a significant main effect of sex [Pillai’s Trace *V* = 0.289, *F*(3,114) = 15.478, *P < *0.001], but not diagnosis [*V* = 0.030, *F*(3,114) = 1.183, *P = *0.32] or their interaction [*V* = 0.017, *F*(3,114) = 0.643, *P = *0.59]. *Post hoc* ANOVAs showed that the main effect of sex was driven by larger volumes in males than females and was evident across grey matter [*F*(1,116) = 35.623, *P < *0.001, 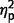 = 0.235], white matter [*F*(1,116) = 38.727, *P < *0.001, 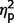 = 0.250] and CSF [*F*(1,116) = 12.464, *P = *0.001, 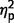 = 0.097].

**Figure 1:**
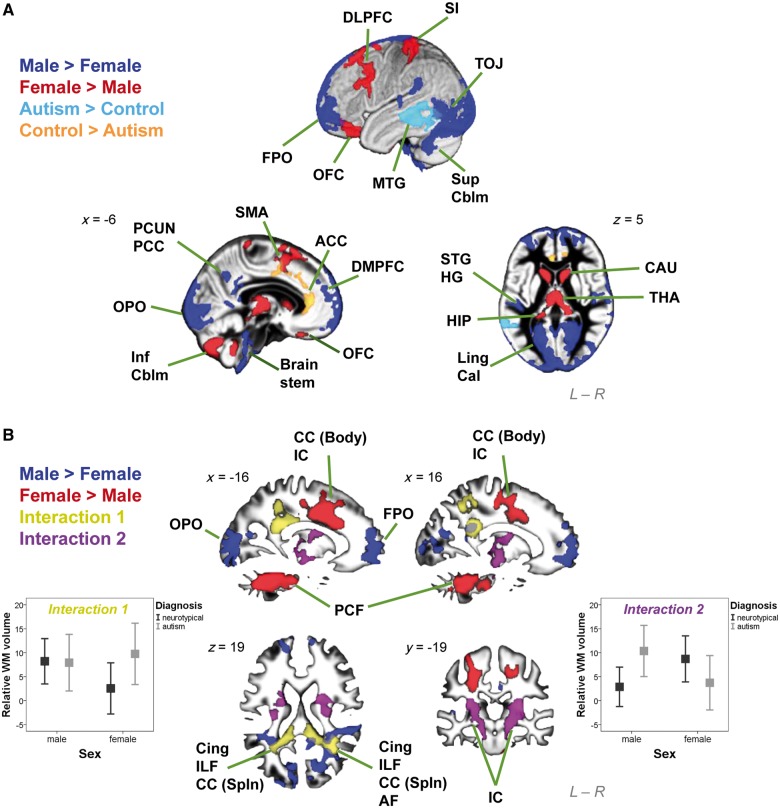
Brain structures showing significant interaction and main effects in the 2 x 2 factorial design VBM. (**A**) Clusters were overlaid on the grey matter segment of the study-specific template. Substantially large clusters where males are larger than females are in dark blue, females larger than males in red, neurotypical controls larger than autism groups in orange, and autism groups larger than neurotypical controls in light blue. (**B**) Clusters were overlaid on the white matter segment of the study-specific template. Substantially large clusters where males are larger than females are in dark blue, and females larger than males in red. Importantly, large clusters with significant sex × diagnosis interactions were noted. A pattern of FA > FC but MA = MC (left error-bar graph; y-axis indicates relative white matter volume [arbitrary unit] and error bar indicates standard error of the mean) was identified in two clusters (yellow), and a pattern of MA > MC but FA < FC (right error-bar graph) in two clusters (purple). ACC = anterior cingulate cortex; AF = arcuate fasciculus; Cal = calcarine; CAU = caudate; CC (Body) = body of corpus callosum; CC (Spln) = splenium of corpus callosum; Cing = cingulum; DLPFC = dorsolateral prefrontal cortex; DMPFC = dorsomedial prefrontal cortex; FPO = frontal pole; HG = Heschl gyrus; HIP = hippocampus; IC = internal capsule; ILF = inferior longitudinal fasciculus; Inf Cblm = inferior cerebellum; Ling = lingual gyrus; MTG = middle temporal gyrus; OFC = orbitofrontal cortex; OPO = occipital pole; PCC = posterior cingulate cortex; PCF = ponto-cerebellar fibres; PCUN = precuneus; SI = primary somatosensory cortex; SMA = supplementary motor area; STG = superior temporal gyrus; Sup Cblm = superior cerebellum; THA = thalamus; TOJ = temporo-occipital junction.

At a local level the 2 × 2 factorial design analysis on grey matter (Fig. 1A) showed substantially large clusters with significant main effects of both sex and diagnosis, but no regions with a significant sex × diagnosis interaction, unless the cluster-forming voxel-level threshold was relaxed from *P < *0.025 to *P < *0.05. Males had larger volume than females in six clusters distributed across the bilateral frontal and occipital poles, dorsomedial prefrontal cortices, sensori-motor cortices, superior temporal gyri, Heschl gyri, lingual and calcarine gyri, temporo-occipital and lateral temporal regions, precuneus, posterior cingulate cortices, superior cerebellar hemispheres and brainstem. Females had larger volume than males in nine clusters involving left dorsolateral prefrontal cortex, supplementary motor area, primary somatosensory cortex, and bilateral orbitofrontal cortices, caudate, thalamus, fusiform, hippocampal and parahippocampal gyri, cerebellar vermis and hemispheres (inferior lobules). Autism groups were larger than neurotypical controls in one cluster involving left middle temporal gyrus. Neurotypical controls were larger than autism groups in one cluster involving bilateral anterior cingulate cortices and supplementary motor areas (Supplementary Table 1). There was no significant correlation between the volumes of regions showing main effects of diagnosis and any of the symptom measures in any of the four groups.

For white matter (Fig. 1B), there were significant main effects of sex but not diagnosis. Males were larger than females in six clusters bilaterally distributed in the frontal, occipital and temporo-parieto-occipital junction regions. Females were larger than males in three clusters, including one in the cerebellum and brainstem, and two bilaterally in posterior frontal lobe involving internal capsule and fibres from the body of corpus callosum. Importantly, significant interactions in both directions were seen, indicating that autism manifests differently according to sex. A pattern of females with autism greater than typical females (FA > FC) but males with autism equal to typical males (MA = MC) was identified in two clusters in bilateral temporo-parieto-occipital regions, involving the posterior portion of bilateral cingulum, inferior longitudinal fasciculus, corpus callosum (splenium) and right arcuate fasciculus [right-lateralized cluster size k_e_ = 11 473 voxels, cluster-level FDR-corrected *q* < 0.001, peak-voxel MNI coordinate (35, −54, 16), *T* = 4.38; left-lateralized cluster k_e_ = 6409, cluster-level *q* = 0.002, peak-voxel (−18, −39, 25) *T* = 3.86]. Another pattern of males with autism greater than typical males (MA > MC) but females with autism smaller than typical females (FA < FC) was identified in two clusters involving internal capsule bilaterally at the level around basal ganglia and thalamus [right-lateralized cluster k_e_ = 7234, cluster-level *q* = 0.003, peak-voxel (32, −21, 7) *T* = 3.56; left-lateralized cluster k_e_ = 5558, cluster-level *q* = 0.008, peak-voxel (−32, −9, 7) *T* = 4.02] (Supplementary Table 2). There was no significant correlation between the volumes of regions showing these sex × diagnosis interaction effects and any of the symptom measures in any of the four groups.

**Figure 2:**
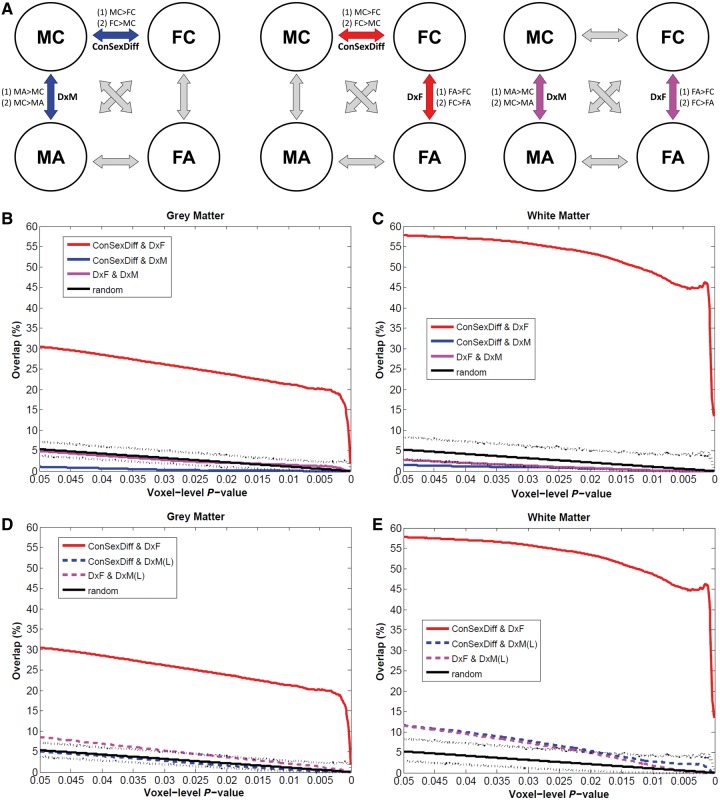
Testing brain-level predictions of the EMB theory of autism. (**A**) The three repeated diagrams illustrate the analytic strategy, measuring spatial overlap between VBM comparisons (double arrows) between two of the four groups (circles). Whether there is a substantial overlap between MC–FC (‘ConSexDiff’) and MC–MA (‘DxM’) tests the EMB theory prediction in males (left diagram, blue arrows, two spatial overlap analyses for two pairs of contrasts [(1) and (2)], each using two VBM group-difference maps); whether there is a substantial overlap between MC–FC and FC–FA (‘DxF’) tests the EMB theory prediction in females (middle diagram, red arrows). Additionally, comparing MC–MA and FC–FA indicates how similar the neuroanatomical features of autism are in males and in females (right diagram, purple arrows). (**B** and **C**) Spatial overlaps of VBM group-difference maps are shown along voxel-level thresholds from *P = *0.05 down to *P = *0.0001 for grey matter (**B**) and white matter (**C**). Red lines indicate the percentage of voxels characterizing diagnostic effect of autism in females (DxF) that also belong to those characterizing sex differences in controls (ConSexDiff), averaged from both directions of contrasts. Blue lines indicate the same for males using the main sample (DxM). Purple lines represent the overlap between voxels characterizing autism in females and that in males (DxF & DxM). Black solid lines indicate the average overlap occurred under random conditions derived from 5000 Monte Carlo simulations, with dotted lines indicating the 0.5 and 99.5 percentiles. Red lines constantly show high values irrespective of the voxel-level threshold, whereas all others are markedly lower and are below or within the random range. (**D** and **E**) These repeat panels **B** and **C**, but using the MA–MC group-difference maps derived from the larger multicentre male sample (*n = *84/group). Red lines (overlap in females) and black lines (random condition) are exactly the same as those in **B** and **C**. Blue and purple dashed lines indicate the same analyses but using the larger male sample [DxM(L)]. These replicate the findings from the main sample.

Spatial overlap between the atypical neuroanatomical features of autism in females and males (Fig. 2A, right) was minimal (e.g. 2.3% for grey matter and 1.0% for white matter in voxel-level *P < *0.025 maps; Fig. 2B and C, purple solid lines) irrespective of the voxel-level threshold, and did not differ from simulations measuring random overlap of clusters (i.e. area between the black dotted lines). This confirms that in brain morphology, males and females with autism differ from same-sex controls in distinct ways. An additional analysis using a larger (*n = *84/group) multicentre male sample replicated this observation (Fig. 2D and E, purple dashed lines).

For VBM comparison between females with and without autism (Supplementary material and Supplementary Fig. 2).

### Question 2: Does the neuroanatomy of autism fit predictions from the EMB theory of autism, in males and/or in females?

Here we tested the extent to which structures sensitive to autism diagnosis are also typically sexually dimorphic. For grey matter, irrespective of the voxel-level threshold, we found no evidence for substantial overlap between structures sensitive to diagnosis in males (MA > MC, MC > MA) and sex differences between controls (MC > FC, FC > MC) under directionality predicted by the EMB theory (Fig. 2B, blue solid line; probability *P* > 0.999, suggesting no overlap between maps; e.g. in voxel-level *P < *0.025 thresholded maps, 0.2% of the autism-control difference map in males overlapped with typically sexually dimorphic structures). The overlap was negligibly higher (e.g. 2.3% in voxel-level *P < *0.025 thresholded maps) irrespective of the voxel-level threshold, when using the within-male group-difference map from the additional VBM analysis carried out on a larger multicentre male sample, and was not different from overlap occurring at random chance (*P* > 0.001) (Fig. 2D, blue dashed line).

In contrast, there was substantial overlap between structures sensitive to diagnosis in females (FA > FC, FC > FA) and sex differences between controls, irrespective of the voxel-level threshold (Fig. 2B, red line). For example, 13.8% (at voxel-level *P < *0.0005) to 25.1% (at voxel-level *P < *0.025) of voxels in the autism-control difference map in females overlapped with sexually dimorphic structures in controls. This was significantly larger than expected under a null hypothesis of random cluster overlap generated by Monte Carlo simulations (*P* < 0.001).

**Figure 3:**
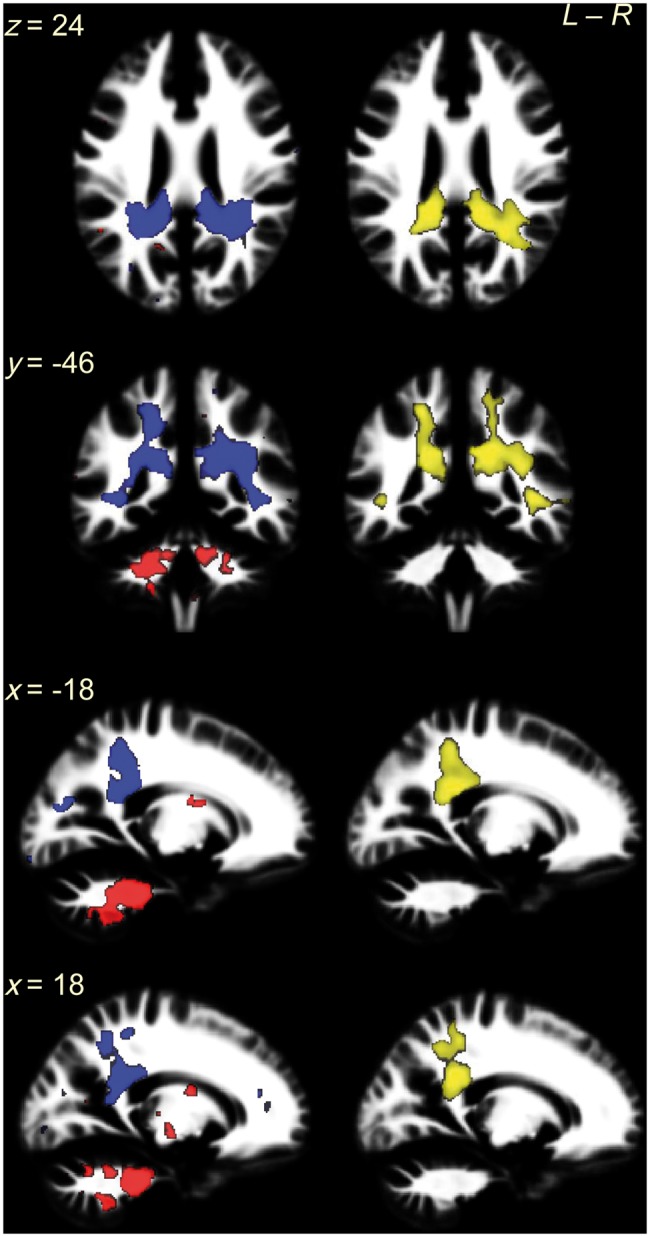
White matter overlapping regions in females coincide with clusters showing a significant sex × diagnosis interaction in the 2 × 2 factorial design. The *left* column shows the white matter overlapping voxels for ‘FC > FA AND FC > MC’ contrasts (red) and ‘FA > FC AND MC > FC’ contrasts (blue) from voxel-level *P < *0.025 maps. The majority of them were spatially contiguous and constituted bilateral clusters: the former (red) involved ponto-cerebellar fibres and the latter (blue) involved cingulum, corpus callosum (splenium), inferior longitudinal fasciculus and arcuate fasciculus. Particularly, the latter located at the same regions as clusters showing a sex × diagnosis interaction in the 2 × 2 factorial design VBM (*right* column, yellow; thresholded at voxel-level *P < *0.025 and corrected for multiple comparisons by ensuring a cluster-wise topological FDR at *q* < 0.05). This replication in location of the overlap (blue, from analysis to Question 2) and sex × diagnosis interaction (yellow, from analysis to Question 1) illustrates the fact that the linkage between neuroanatomical features of autism and features of typical sexual dimorphism is specific to females, because this linkage is statistically significantly different from that in males, who lack such a linkage.

Even clearer results were noted for white matter. The overlap between structures sensitive to autism diagnosis in males and sexually dimorphic structures in controls was again minimal in the main and the larger samples (e.g. 0.9% and 7.7%, respectively in voxel-level *P < *0.025 maps; Fig. 2C, blue solid line, and E, blue dashed line). Strikingly however, the overlap in females (Fig. 2C, red line) was extensive (e.g. 24.5% in voxel-level *P < *0.0005 maps and 55.3% in *P < *0.025 maps) and occurred non-randomly (*P* < 0.001), irrespective of the voxel-level threshold. An example of such overlap included most voxels showing a sex × diagnosis interaction in the earlier 2 × 2 factorial analysis (Fig. 3), reconfirming that only in females, but not in males, there is a close relationship between the neuroanatomy of autism and neural sexual dimorphism in controls.

In sum, we observed marked sex differences within autism in terms of neuroanatomy. Atypical features in females, but not males, overlapped with areas showing typical sexual dimorphism in controls, confirming predictions from the EMB theory in females but not in males.

### Are brain regions that fit predictions from the extreme male brain theory correlated with prenatal sex steroid hormone influence?

Prenatal sex steroids are one biological mechanism shaping early brain development and the emergence of sexual dimorphism (Lombardo *et al.*, 2012*a*, *b*). One proxy measure for prenatal sex steroid influence is the 2D:4D ratio, where a lower ratio between the second and fourth digits indicates higher androgen compared with oestrogen influence (Zheng and Cohn, 2011). Therefore, we tested if the brain regions in females that fit EMB theory predictions correlate with 2D:4D ratio.

**Figure 4:**
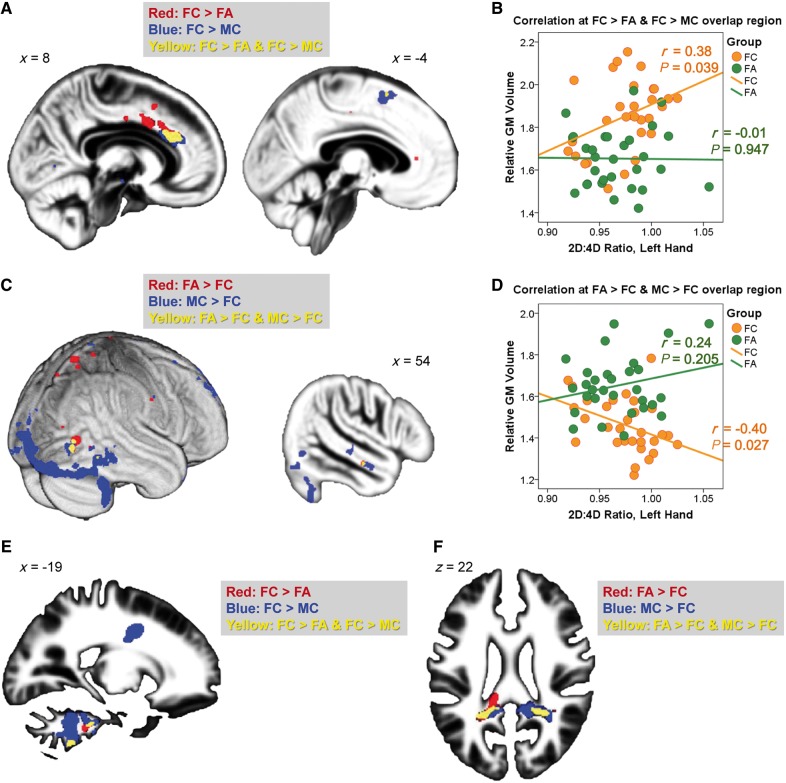
Overlapping region and 2D:4D ratio. (**A–D**) Overlapping grey matter regions (yellow, from voxel-level *P < *0.0005 maps) linking neuroanatomical features of autism in females (FC–FA) to those of sex differences in control subjects (MC–FC) were spatially contiguous and showed a volumetric correlation to left-hand 2D:4D ratio in neurotypical females but not females with autism, for both directions of contrasts [**A**: overlap at right anterior cingulate cortex (*left*) and a smaller cluster at left supplementary motor area (*right*); **B**: the correlations for regions in **A**; **C**: overlap at right extrastriate visual cortex (*left*) and middle temporal gyrus (*right*); **D**: the correlations for regions in **C**]. These regions reflect neuroanatomical features of autism in females, show typical sex differences, and have volumetric correlations to prenatal sex hormonal processes only in neurotypical females, possibly because their volumes in females with autism were already close to the limits for females in general, evidenced by the convergence of the regression lines of the two groups under strongest prenatal androgen effects (i.e. lowest 2D:4D ratio). (**E** and **F**) Overlapping white matter regions were spatially contiguous and involved the ponto-cerebellar fibres (**E**) and posterior corpus callosum, cingulum, inferior longitudinal fasciculus and arcuate fasciculus (**F**). They failed to show a volumetric correlation with 2D:4D ratio. GM = grey matter.

Overall, females with or without autism in this sample did not differ in their left-hand [females with autism: mean = 0.967, standard deviation SD = 0.0322; female control subjects: mean = 0.975, SD = 0.0287; *t*(58) = 1.010, *P = *0.317] or right-hand [females with autism: mean = 0.971, SD = 0.0256; female control subjects: mean = 0.972, SD = 0.0293; *t*(58) = 0.171, *P = *0.865] 2D:4D ratios. However, on examination of regions fitting EMB theory predictions (identified from the overlap analyses, at a conservative voxel-level threshold of *P < *0.0005) we found a positive correlation between left-hand 2D:4D ratio and relative grey matter volume of the ‘FC > FA AND FC > MC’ overlapping voxels (Fig. 4A and B) in right anterior cingulate cortex and left supplementary motor area in neurotypical females (*r* = 0.38, *P = *0.039), but not females with autism (*r* = −0.01, *P = *0.947). There was a trend toward significance in the difference between these correlations (*P = *0.088). We also observed a negative correlation between left-hand 2D:4D ratio and relative grey matter volume of the ‘FA > FC AND MC > FC’ overlapping voxels (Fig. 4C and D) in the right extrastriate visual cortex and middle temporal gyrus in neurotypical females (*r* = −0.40, *P = *0.027), but not females with autism (*r* = 0.24, *P = *0.205). The difference between these correlations was significant (*P = *0.014). These results suggest that in neurotypical females, regions fitting EMB theory predictions are sensitive to prenatal sex steroid influence. Such correlations, however, were not found to be significant in females with autism.

For white matter overlapping regions (Fig. 4E and F), none of the correlations with left-hand 2D:4D ratio were significant. The right-hand 2D:4D ratio was not significantly correlated with volume of any grey matter/white matter overlapping regions.

These correlation analyses (eight in total) were exploratory, therefore the significance level was not adjusted for multiple comparisons. In addition, the selection of regions for testing and actual testing were performed on the same data, thus the magnitude of effect sizes might not reflect the true effect size.

## Discussion

We found evidence suggesting that aspects of the neuroanatomy of autism in high-functioning adults are sex-dependent. Furthermore, only females with autism show atypical neuroanatomical features that substantially overlap with structures showing sexual dimorphism in neurotypical controls (i.e. neuroanatomical ‘masculinization’), indicating that predictions from the EMB theory of autism are observed in females, but not in males.

### At the neural level, females and males with autism may comprise different subgroups

Our first aim was to test if there is a substantially large cluster (spatial-level test) showing significant sex × diagnosis interaction (magnitude-level test) in the 2 × 2 factorial design VBM. Such interactions were clear in white matter (but marginal in grey matter), suggesting that some neuroanatomical features of autism manifest differently by sex. These observations extend previous work from smaller studies showing sex × diagnosis interaction on white matter integrity (in corpus callosum, cingulum and corona radiata) and grey matter volume (at the right inferior parietal lobe and rolandic operculum) (Beacher *et al.*, 2012*a*), and in brain activation during a mental rotation task (Beacher *et al.*, 2012*b*).

Within the present sample we also found that there was minimal overlap between the neuroanatomical features of autism in males and females (Fig. 2). The neuroanatomical features of females with autism (Supplementary Fig. 2) substantially replicate observations from a previous smaller study (Craig *et al.*, 2007), and furthermore, both findings are distinct from areas found in studies of males (or majority male samples) as revealed in the largest meta-analyses to date (Radua *et al.*, 2011; Via *et al.*, 2011). The converging message is that for high-functioning adults with autism, males and females may have different structural neurophenotypes.

It has been proposed that, if females are innately less vulnerable to developing autism, more ‘severe’ brain changes may be necessary for them to reach the point of clinical diagnosis (Wing, 1981; Lord *et al.*, 1982; Craig *et al.*, 2007; Murphy *et al.*, 2011). If true, we should expect that: (i) females with autism should show larger effect sizes in changes relative to neurotypical females, in the same regions that differ between males with and without autism; and/or (ii) females with autism should have broader spatial involvement, including and transcending the regions that are atypical in males with autism. Surprisingly, neither of these predictions were supported by our findings or by other recent reports (Craig *et al.*, 2007; Calderoni *et al.*, 2012). Although there is a long-held view that females with autism tend to be more severely affected cognitively (and potentially neurally) (Tsai *et al.*, 1981; Wing, 1981; Lord *et al.*, 1982; Tsai and Beisler, 1983; Lord and Schopler, 1985), our data on high-functioning adults do not confirm such a picture. One explanation is that general cognitive ability further affects how autism manifests in males and females. It may be that high-functioning females with autism are neurally and cognitively ‘different from’ rather than ‘more severe than’ their male counterparts. It is also possible that in the more disabled population, females with autism are indeed more neurally and cognitively impaired than males with autism. This awaits future studies employing a similar design as ours.

The sex-specific neuroanatomy discovered here for high-functioning adults with autism are in line with the growing evidence of sex-specific biological profiles for the high-functioning subgroup at the levels of serum proteomics (Schwarz *et al.*, 2011; Ramsey *et al.*, 2012), sex steroid hormones and anthropometry (Ruta *et al.*, 2011; Bejerot *et al.*, 2012), and for the whole autism spectrum at the levels of genetics (Gilman *et al.*, 2011; Puleo *et al.*, 2012; Szatmari *et al.*, 2012), transcriptomics (Kong *et al.*, 2012) and early brain overgrowth (Sparks *et al.*, 2002; Bloss and Courchesne, 2007; Schumann *et al.*, 2009, 2010; Nordahl *et al.*, 2011). In sum, high-functioning males and females with autism, though diagnosed by the same behavioural criteria, differ in aspects of neuroanatomy. Testing the mechanisms (aetiological, developmental and experiential) accounting for this will help clarify the male-liability and sources of heterogeneity in autism (Werling and Geschwind, 2013).

### Brain-level predictions of the extreme male brain theory of autism are observed in females but not in males

Our second aim was to test predictions from the EMB theory of autism at the level of neuroanatomy. The theory, in its original formulation at the cognitive level (Baron-Cohen, 2002), did not address whether males and females with autism may be different, but equally did not exclude the possibility that ‘masculinization’ may be expressed differently in each sex. By examining males and females separately, we found that females with autism had neuroanatomical features that overlapped substantially with sexually dimorphic structures in controls. In males with autism, EMB predictions at the neural level were not confirmed. Owing to the study design (cross-sectional and on adults), these findings cannot provide direct aetiological or developmental accounts for autism. However, three competing interpretations may offer insights for future studies in how physiological mechanisms associated with sexual differentiation may have an impact on the development of autism, in males and females, respectively.

The first interpretation is that the findings provide partial support for the EMB theory, in a sex-specific manner, at the neuroanatomical level. Given that the emergence of typical sexual dimorphism in brain structure reflects both sex chromosome and sex hormonal effects (Baron-Cohen *et al.*, 2011; McCarthy and Arnold, 2011), this female-specific observation suggests that these physiological factors related to sexual differentiation may be critical for females but not for males with autism. For example, if sex hormones are involved, sex-specific effects might be reflective of non-monotonic dose-responses and low-dose effects; small variation in hormone dosage can have larger effects at lower doses, which may explain more pronounced effects at the level of neuroanatomy in females compared with males (Vandenberg *et al.*, 2012). A more ‘typically male’-like behavioural (Ingudomnukul *et al.*, 2007; Knickmeyer *et al.*, 2008) and physiological profile (Knickmeyer *et al.*, 2006; Ingudomnukul *et al.*, 2007; Ruta *et al.*, 2011; Schwarz *et al.*, 2011; Bejerot *et al.*, 2012) has been repeatedly noted for females with autism. However, an extreme ‘typically male’ profile for males with autism is less consistently found. Although some studies show an ‘extreme-male’ pattern in high-functioning male adults with autism in serum androstenedione level (Ruta *et al.*, 2011) and in functional MRI response during affective social decision-making (Hall *et al.*, 2012), others do not find such ‘extreme-male’ physiological profiles (Schwarz *et al.*, 2011; Bejerot *et al.*, 2012). How physiological factors related to sexual differentiation may be mechanistically associated with different levels of autistic characteristics, in males and females, respectively, should be a major research question.

Biologically, both sex chromosome (Lee *et al.*, 2012) and sex hormonal effects (Baron-Cohen *et al.*, 2011) are likely to be contributors to the current observations. Although we were unable to examine sex chromosome effects in the current design, we did find some preliminary evidence that sensitivity to prenatal sex steroid influence (indexed by the 2D:4D ratio) may be relevant for regions (e.g. anterior cingulate cortex, extrastriate cortex) that fit predictions from the EMB theory. The anterior cingulate cortex possesses a high density of sex steroid receptors in primates during early development (Clark *et al.*, 1988), and thus may be sensitive to prenatal androgens. Prenatal androgens contribute significantly to brain masculinization (Hines, 2003) and correlate with cognitive traits relevant to autism (e.g. rate of language development, eye contact, empathy, systemizing, and attention to detail) in typically developing children (Baron-Cohen *et al.*, 2011). It is thus plausible that prenatal sex steroid hormones influence neurodevelopment related to autism, particularly in females.

A second interpretation to the female-only finding, assuming the original EMB theory holds, is that although physiological factors related to sexual differentiation may be critical for autism, ceiling effects in males may have obscured our ability to detect similar effects in males. That is, if the typically developing male brain has already approached a limit in terms of volumetric ‘masculinization’, it would be difficult to detect if males with autism are even more extreme. As females are not at ceiling, small physiological variations may produce more easily observable effects on brain volume. This may apply not only to the neuroanatomical but also to other physiological and behavioural aspects reviewed earlier.

In keeping with this ceiling effect assumption, the lack of a significant volumetric correlation with 2D:4D ratio in females with autism may occur under a similar logic: they may have already been ‘masculinized’ to the range approaching the limit for females, rendering the correlation less easily detectable than that in neurotypical females. The convergence of regression lines at the lowest point for the 2D:4D ratio in Fig. 4B and D supports this interpretation. Alternatively, the differential correlation in females with and without autism may be attributable to other factors modulating neurodevelopmental effects of prenatal sex steroid hormones, rather than simply the amount of stimulation. The absence of a group difference in 2D:4D ratio corresponds with this view.

A final interpretation is that the observations in fact result from factors unrelated to sexual differentiation. The previous two interpretations assume that typical brain sex differences are the product of physiological mechanisms associated with sexual differentiation. However, variability in brain volume might instead be a product of experiential mechanisms (e.g. gendered experiences; Cheslack-Postava and Jordan-Young, 2012) or biological mechanisms unrelated to sexual differentiation (e.g. genetic or epigenetic effects that are not sex-linked). If true, the observed confirmation to EMB theory predictions is just a ‘neural phenocopy’, arising from these additional mechanisms not associated with sex chromosome or sex hormonal effects. Careful studies are needed to dissect these different, though not mutually exclusive, interpretations.

### An alternative hypothesis linking autism to biological sex differences: Are males with autism ‘feminized’ in terms of neuroanatomy?

On the theoretical side, it is worth noting that the EMB theory is not the only hypothesis predicting a relationship between autism and biological sexual differentiation. Bejerot *et al.* (2012) proposed that autism is associated with ‘gender incoherence’ or androgyny at the physiological domain, and these claims were supported by the findings that females with autism have certain ‘masculinized’ physical and biological features, but males with autism are instead ‘feminized’. The two theories’ predictions at the neuroanatomical level are the same for females but different for males with autism. The main neuroanatomical findings for females in the present study fit both theories.

Based on this, we further performed a subsidiary analysis exploring if males with autism, compared with neurotypical males, showed a ‘feminized’ neuroanatomy compatible with the predictions of the ‘gender incoherence’ hypothesis (rather than ‘over-masculinized’ as predicted by the EMB theory). To test this we examined the spatial overlap between MA–MC (using the larger multicentre male data set) and MC–FC maps. Instead of measuring the overlap following the directionality predicted by the EMB theory (MA > MC AND MC > FC, MC > MA AND FC > MC; i.e. how the effect of autism overlaps with the effect of ‘masculinization’), we tested how the effect of autism overlaps with the effect of ‘feminization’ (MA > MC AND FC > MC, MC > MA AND MC > FC).

**Figure 5:**
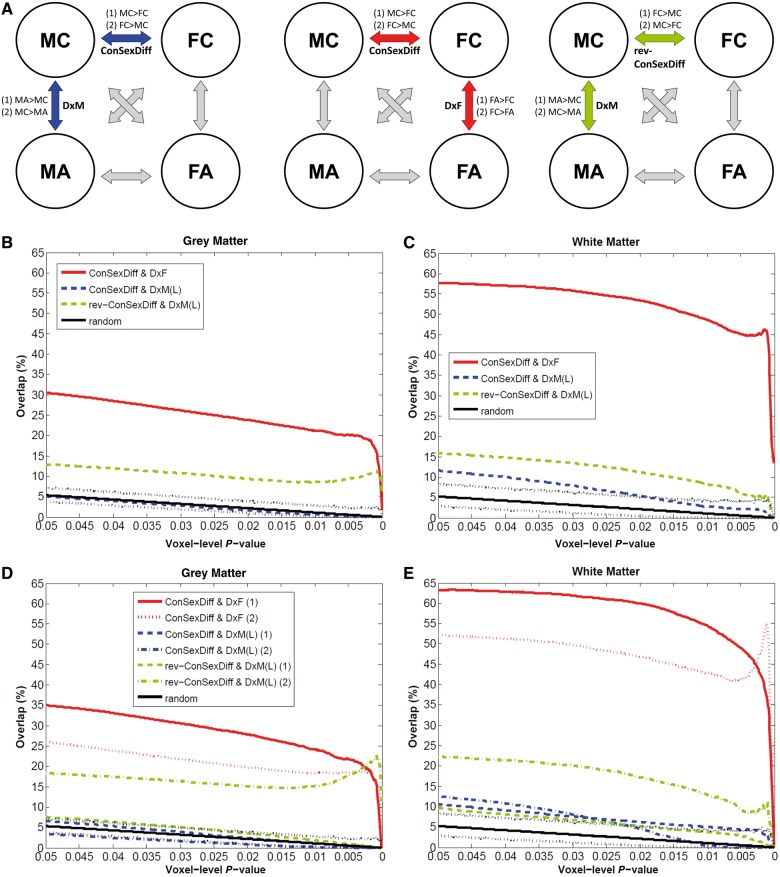
Overlap analyses testing whether males with autism are ‘feminized’ in terms of neuroanatomy. Following Fig. 2, this figure additionally illustrates whether males with autism show neuroanatomical features that resemble typical sex differences but in a direction indicative of ‘feminization’ (**A**, *right*). Green lines indicate the percentage of voxels characterizing the diagnostic effect of autism in males [DxM(L), using the larger multicentre male sample] that also belong to those characterizing ‘feminization’ (rev-ConSexDiff), averaged from both directions of contrasts in panels **B** and **C**, but separately in panels **D** and **E** (**E** shares the same legend as **D**). In panels **B** and **C**, red and blue lines exactly repeat those in Fig. **2****D** and **2****E**. In panels **D** and **E**, for each overlap analysis results from the two directions of pair of contrasts are separately shown.

The results showed that there was a non-random (*P* < 0.001) overlap between structures sensitive to autism diagnosis in males and sexually dimorphic structures in controls representing ‘feminization’, and this was consistently observed across almost all voxel-level thresholds apart from the low *P*-values for white matter (Fig. 5B and C, green dashed lines). However, when further examining the two directions of pair of contrasts separately, it showed that only one of them (‘MC > MA AND MC > FC’, Fig. 5D and E, green dash-dot lines) consistently showed non-random overlap, in both grey and white matter, whereas for the other direction (‘MA > MC AND FC > MC’, Fig. 5D and E, green dashed lines) overlap lay within the range of that observed under random conditions. These results in males with autism stand in sharp contrast to the results observed for females, where both directions of contrasts consistently showed non-random overlap (Fig. 5D and E, red solid and dotted lines).

Together, these neuroanatomical findings may be preliminary and modest evidence that males with autism show ‘gender-incoherence’. However, it is still unclear how this coincides with the cognitive level findings where the data fit predictions from the EMB theory (Supplementary Fig. 1). The relationship among physiology, neuroanatomy and cognition in males and females with autism, respectively, remains an open question for future research.

### Limitations and future directions

Given the heterogeneity in autism, whether the observed sex-dependent neuroanatomy is sample-specific requires replication in other high-functioning samples and other subgroups on the spectrum (e.g. with low IQ and/or with major comorbidity). To explore this within the present sample we performed a split-half validation (Supplementary material and Supplementary Fig. 3). Several other limitations also need to be acknowledged. First, this study is cross-sectional, correlational and focuses on adults so cannot address aetiological and developmental effects. How experiential influences and biological mechanisms related to sexual differentiation that exert early organizational (e.g. sex chromosomal and prenatal sex hormonal effects) or late activational effects (e.g. developmental changes during puberty) interact and contribute to the present findings require future longitudinal studies.

Second, as the participants are of average to above-average IQ and without major comorbidity, it is unknown if the results would generalize to those with lower IQ and/or major comorbidity. Equally, it will be important to examine whether the current findings are characteristic of participants with more severe, explicit and/or clinically significant current autistic symptoms (e.g. those scoring well above the cut-off for ‘autism’ on the Autism Diagnostic Observation Schedule). The variability in cognitive ability and clinical presentation of our participants with autism (particularly females) is not sufficient to be representative of the whole population of individuals ‘on the spectrum’. Therefore, the findings should not be generalized to all individuals with autism, which requires further studies on different subgroups to examine.

Third, although we found little evidence suggesting that high-functioning males and females with autism have substantial shared atypical neuroanatomy, this should not be interpreted as suggesting that males and females with autism are completely different from each other. It may be that effect sizes are smaller in males, so require much larger sample sizes to detect, and such atypical features turn out to be shared by both sexes. In addition, although the method of VBM detects local volumetric differences, volumetric measures are a function of geometric component features such as cortical thickness and surface area (Ecker *et al.*, 2013). Further work should look at sex-general and sex-specific effects within these component features.

Fourth, although one novelty of this study is its ability to answer the two research questions within the same design, an ideal test of EMB theory predictions would use independent group-difference comparisons. That is, one would ideally want to have a large independent sample of neurotypical males and females to define sexually dimorphic regions and a second large independent sample of males and females with and without autism to define regions where diagnosis effects occur.

Fifth, there are also limitations surrounding the exploratory inferences to sex steroid hormones and autism as 2D:4D ratio only explains a small amount of variance of prenatal sex hormone effects (Breedlove, 2010), and the observed volumetric correlation should be interpreted conservatively because it only occurred in the left hand and was not significant for white matter. In addition, although we found group-level neuroanatomical differences and certain group-level behavioural differences (Table 1), we did not find any evidence of linear associations between such neuroanatomical features and behavioural measures at the individual level. These results likely signal the substantial complexity in understanding structure-function relationship when moving from group- towards individual-level, and warrants further investigations.

Lastly, a conceptual obstacle needs to be acknowledged in interpreting the findings for all studies on sex differences in behaviourally defined neuropsychiatric conditions where the behavioural presentations themselves may be partly dependent on sex/gender. In the context of a lack of non-behavioural diagnostic definition of autism, one may argue that, assuming there is a biological markers/definition yet unidentified, males and females may require different behavioural diagnostic criteria due to: (i) plausible qualitative differences in behaviours (Kopp and Gillberg, 2011); (ii) quantitative differences in the sex/gender-specific norms of the distribution of autistic traits (Lai *et al.*, 2013); (iii) plausible developmental differences in behaviours (Lai *et al.*, 2011); and (iv) diagnostic bias of clinicians in real-world settings (Russell *et al.*, 2011). However, it is impossible to discover potential biological markers/definition of autism for both sexes without adopting certain working behavioural definitions of autism, which may or may not take into account one or several of these issues. Therefore, study findings need to be interpreted in the context of the working definition. For this study, the working definition is currently most commonly adopted, based on real-world clinical diagnosis according to Diagnostic and Statistical Manual of Mental Disorders/ International Classification of Diseases criteria without considering qualitative or quantitative differences between sexes; however, developmental differences were considered as we required a definite childhood presentation of autism (via the Autism Diagnostic Interview-Revised) for both sexes. The advantage of this approach is that it reveals findings from males and females with autism defined according to current common clinical practice. The downside is that it is difficult to infer what the findings will be if the behavioural criteria of autism are modified qualitatively or quantitatively by sex/gender. These can be answered only when future studies apply and compare the findings from different working behavioural definitions of autism adjusted qualitatively and/or quantitatively by sex/gender.

We conclude that high-functioning males and females with autism, though diagnostically defined with identical criteria, should not be assumed to be similar at the neuroanatomical level. In females but not males with autism there is evidence suggestive of neuroanatomical ‘masculinization’. How differences in neuroanatomy relate to similarities in cognition between males and females with autism (Lai *et al.*, 2012*b*) remains to be understood. Future research should stratify by biological sex to reduce heterogeneity and to provide greater insight into the neurobiology of autism.

## Supplementary Material

Supplementary Data

## Abbreviations

EMB: extreme male brain
VBM: voxel-based morphometry
FA: females with autism
FC: female neurotypical control subjects
MA: males with autism
MC: male neurotypical control subjects

## Appendix 1

The Medical Research Council Autism Imaging Multicentre Study Consortium (MRC AIMS Consortium) is a UK collaboration between the Institute of Psychiatry (IoP) at King’s College, London, the Autism Research Centre, University of Cambridge, and the Autism Research Group, University of Oxford. The Consortium members are in alphabetical order: Anthony J. Bailey (Oxford), Simon Baron-Cohen (Cambridge), Patrick F. Bolton (IoP), Edward T. Bullmore (Cambridge), Sarah Carrington (Oxford), Marco Catani (IoP), Bhismadev Chakrabarti (Cambridge), Michael C. Craig (IoP), Eileen M. Daly (IoP), Sean C. L. Deoni (IoP), Christine Ecker (IoP), Francesca Happé (IoP), Julian Henty (Cambridge), Peter Jezzard (Oxford), Patrick Johnston (IoP), Derek K. Jones (IoP), Meng-Chuan Lai (Cambridge), Michael V. Lombardo (Cambridge), Anya Madden (IoP), Diane Mullins (IoP), Clodagh M. Murphy (IoP), Declan G. M. Murphy (IoP), Greg Pasco (Cambridge), Amber N. V. Ruigrok (Cambridge), Susan A. Sadek (Cambridge), Debbie Spain (IoP), Rose Stewart (Oxford), John Suckling (Cambridge), Sally J. Wheelwright (Cambridge), and Steven C. Williams (IoP).
