# Supplementary material for: Biological sex affects the neurobiology of autism
Source: Brain. 2013 Aug 9;136(9):2799–815. doi: 10.1093/brain/awt216 (PMC3754459; doi:10.1093/brain/awt216)
Supplement: Supplementary Data [file supp_136_9_2799__index.html]

Biological sex affects the neurobiology of autism — Biological sex affects the neurobiology of autism — Supplementary Data 

# Biological sex affects the neurobiology of autism

## 

files

**Files in this Data Supplement:**

- Supplementary Data - pdf file
- Supplementary Data - pdf file
- Supplementary Data - pdf file
- Supplementary Data - doc file
- Supplementary Data - doc file
- Supplementary Data - doc file
