# Supplementary figures and images for: Biological sex affects the neurobiology of autism
Source: Brain. 2013 Aug 9;136(9):2799–815. doi: 10.1093/brain/awt216 (PMC3754459; doi:10.1093/brain/awt216)

A

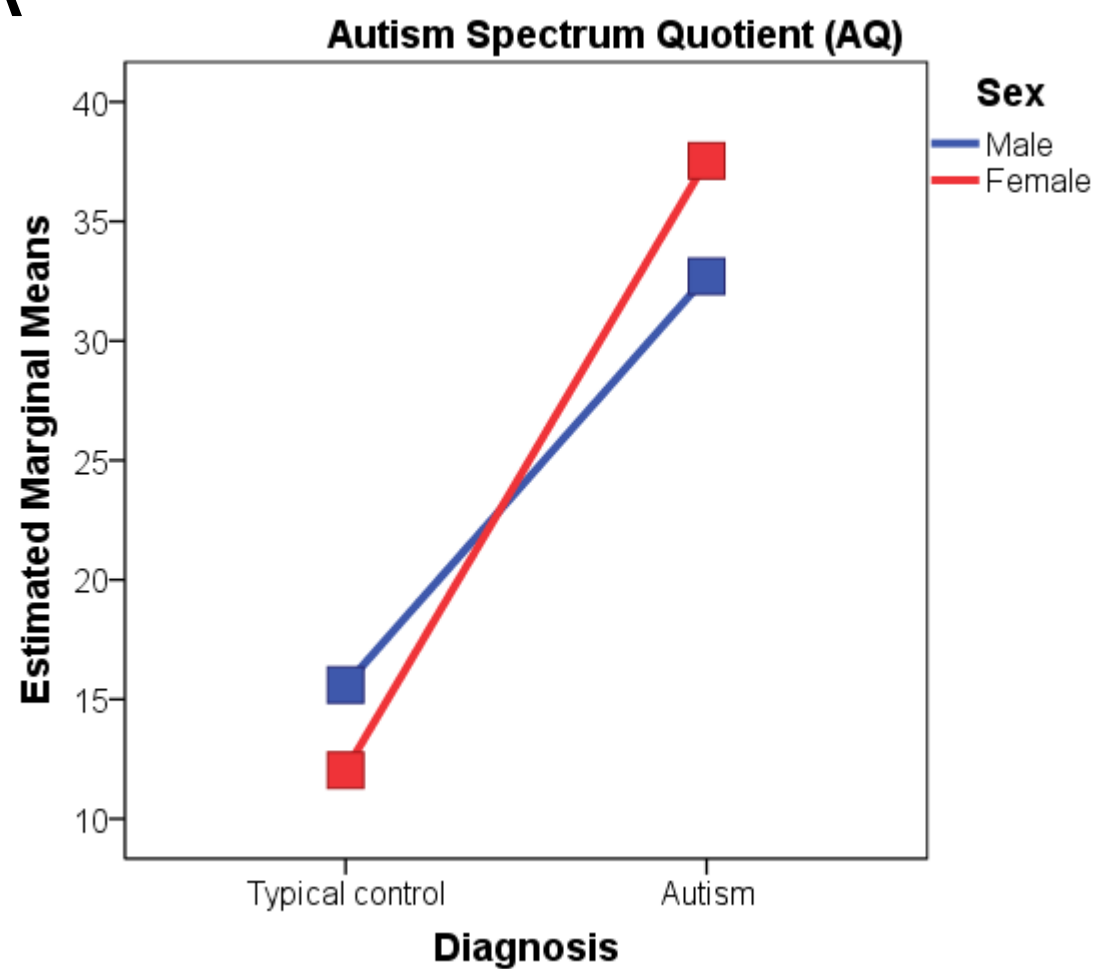

B

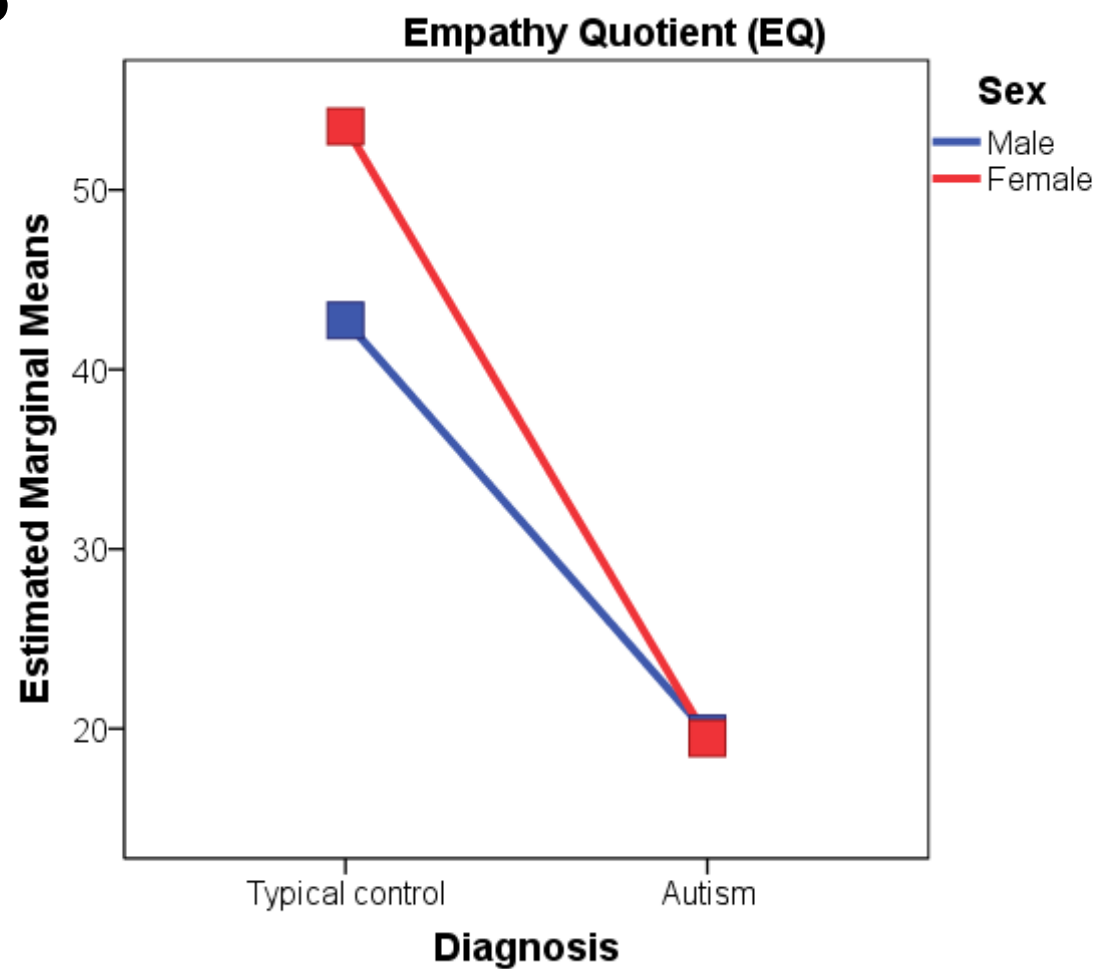

C

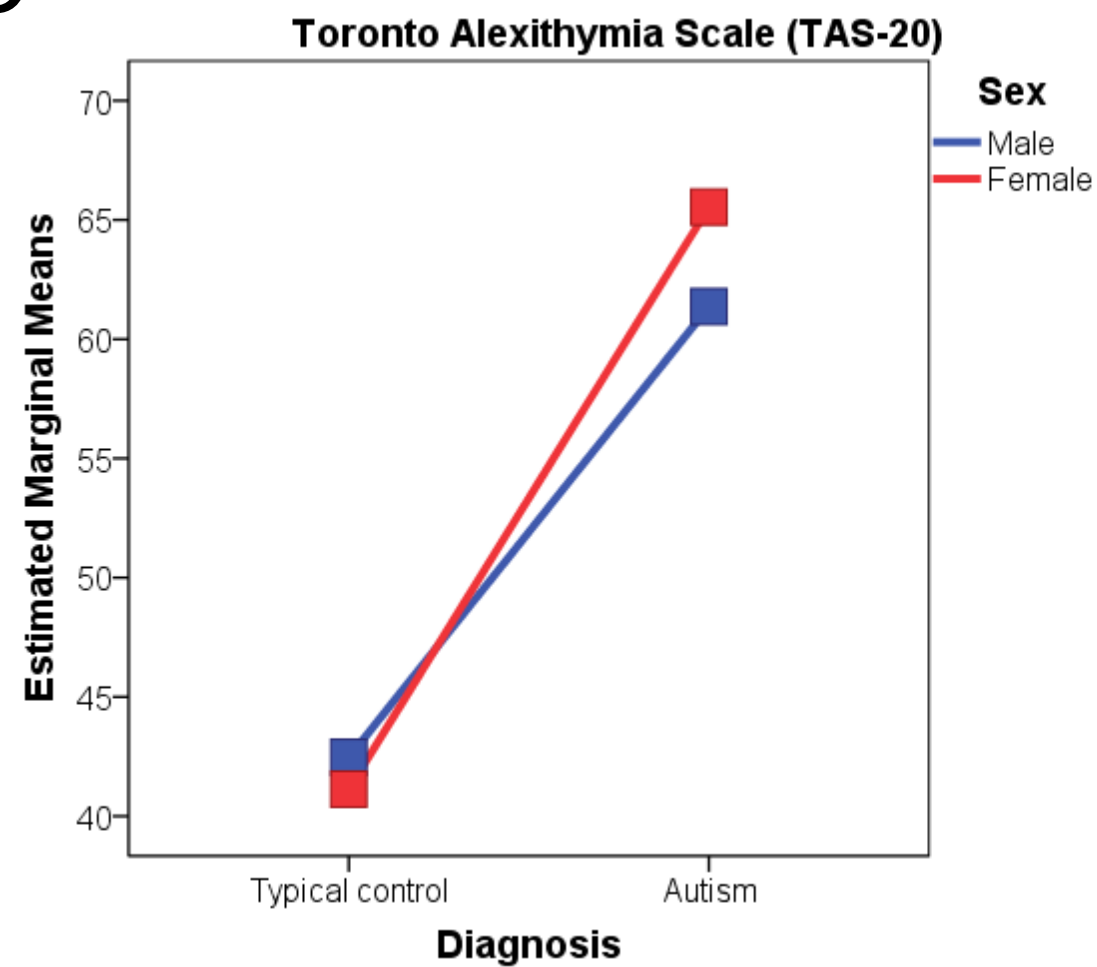

D

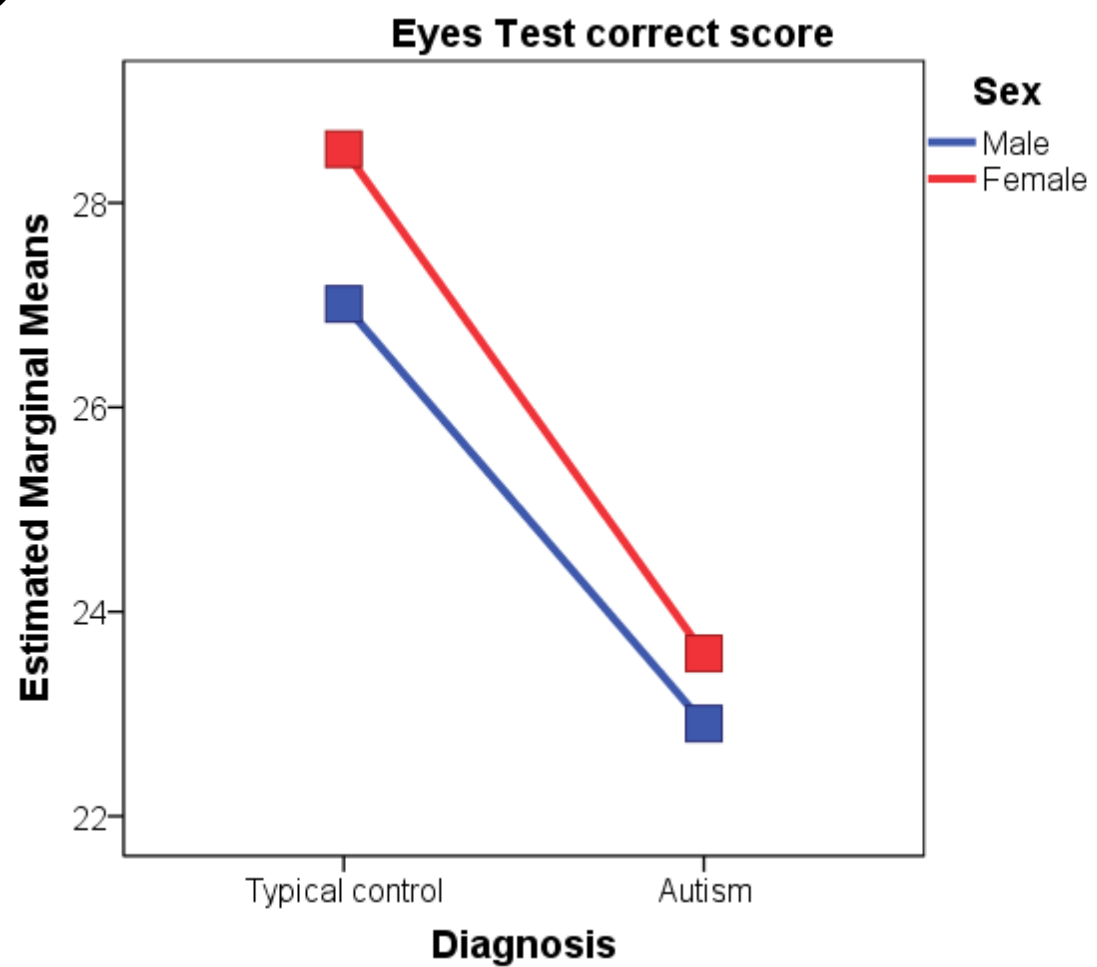

Supplement: Supplementary Data [file supp_awt216_brain-2013-00261-File010.pdf]

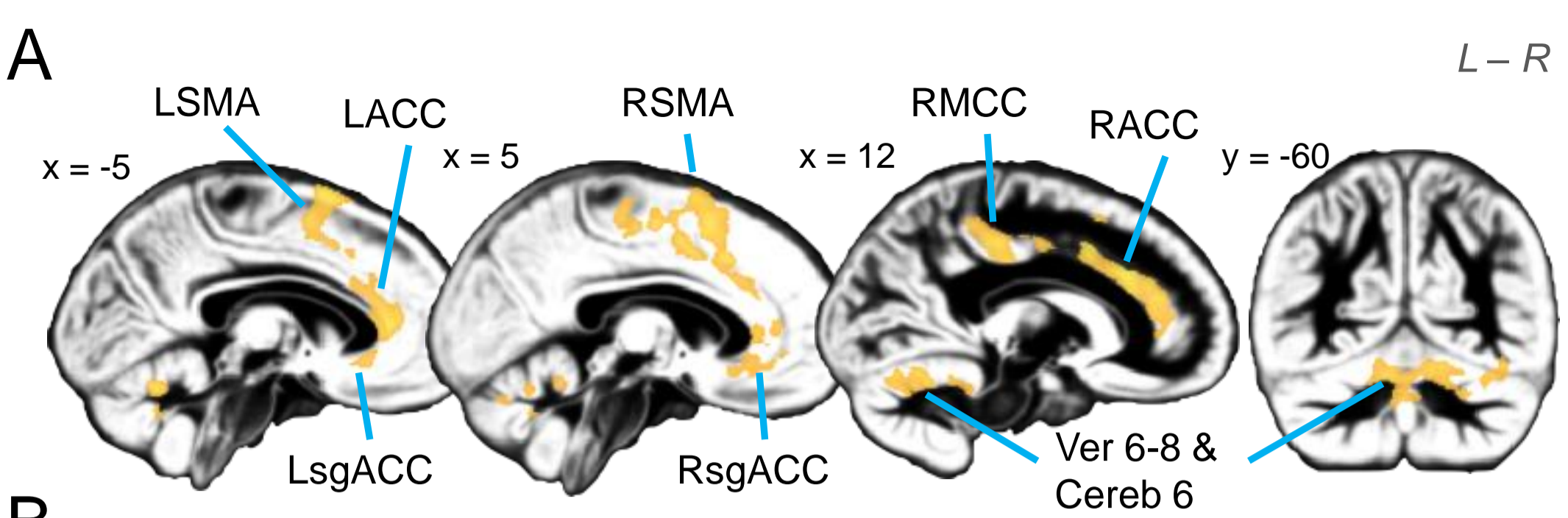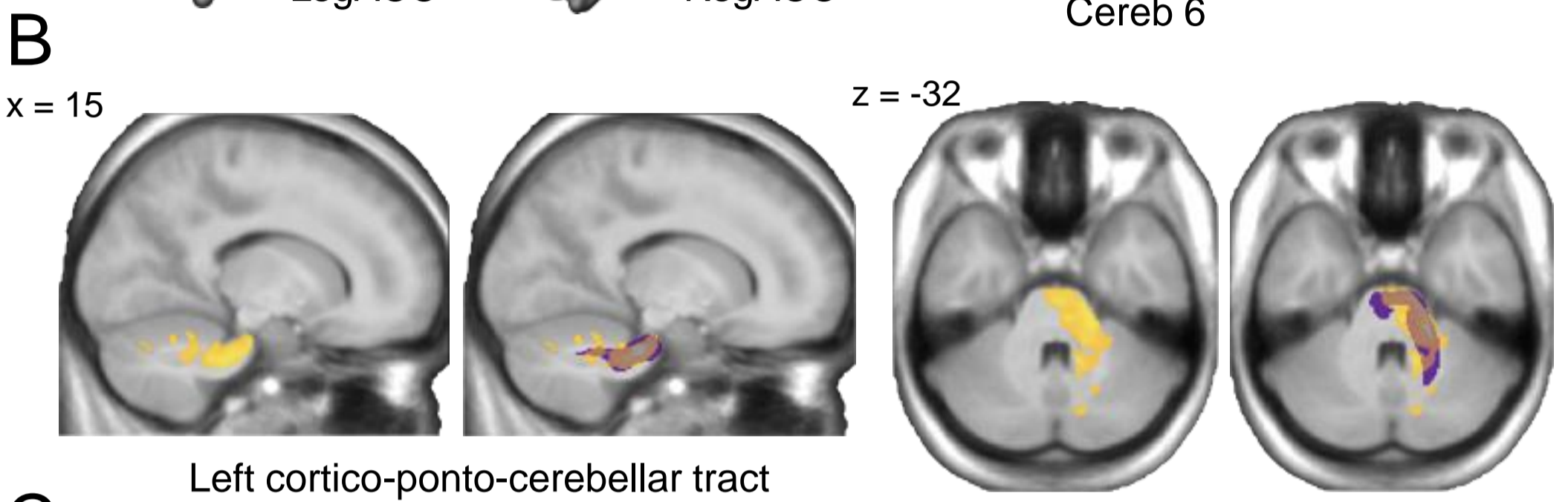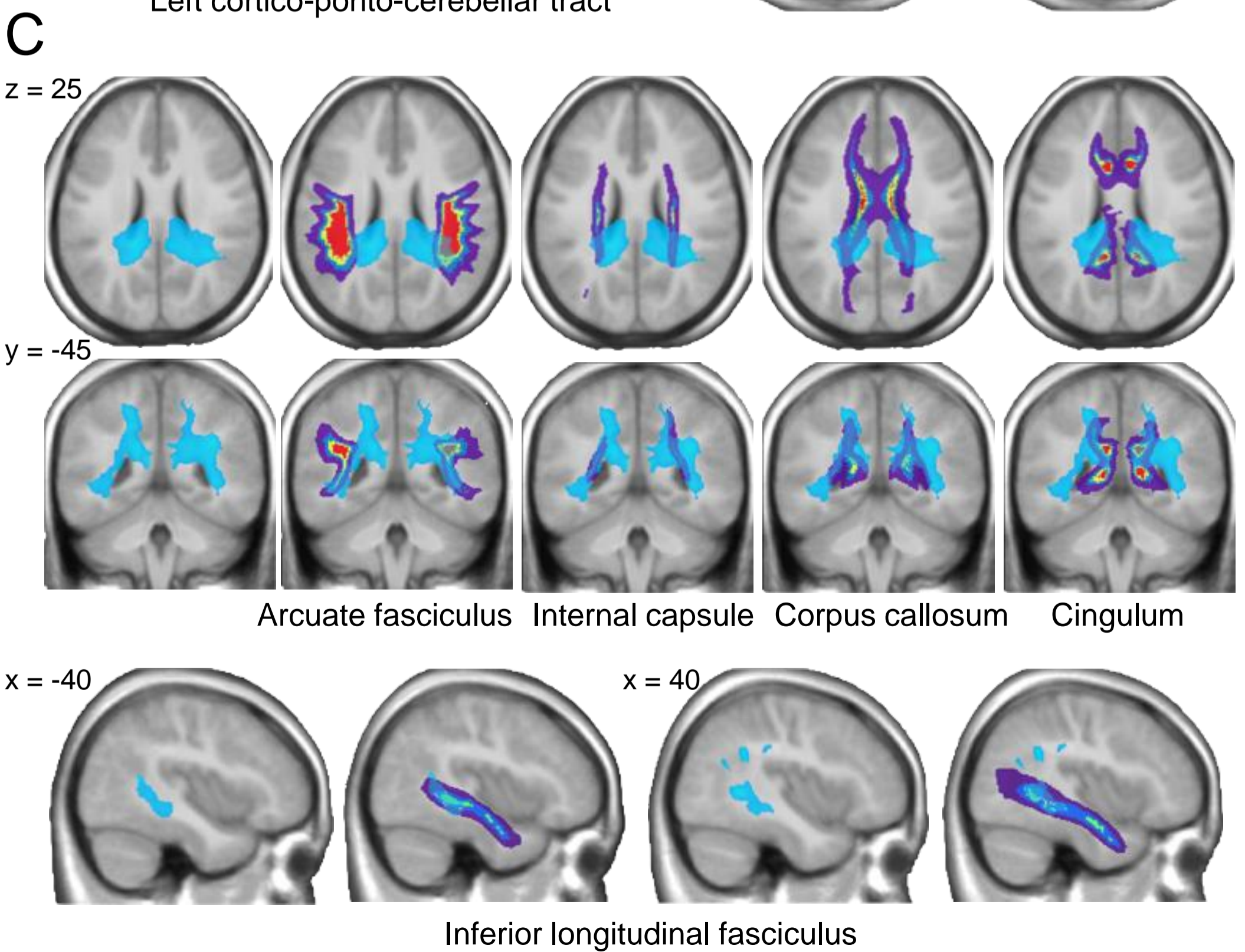

Supplement: Supplementary Data [file supp_awt216_brain-2013-00261-File011.pdf]

A

all

split1

split2

GM

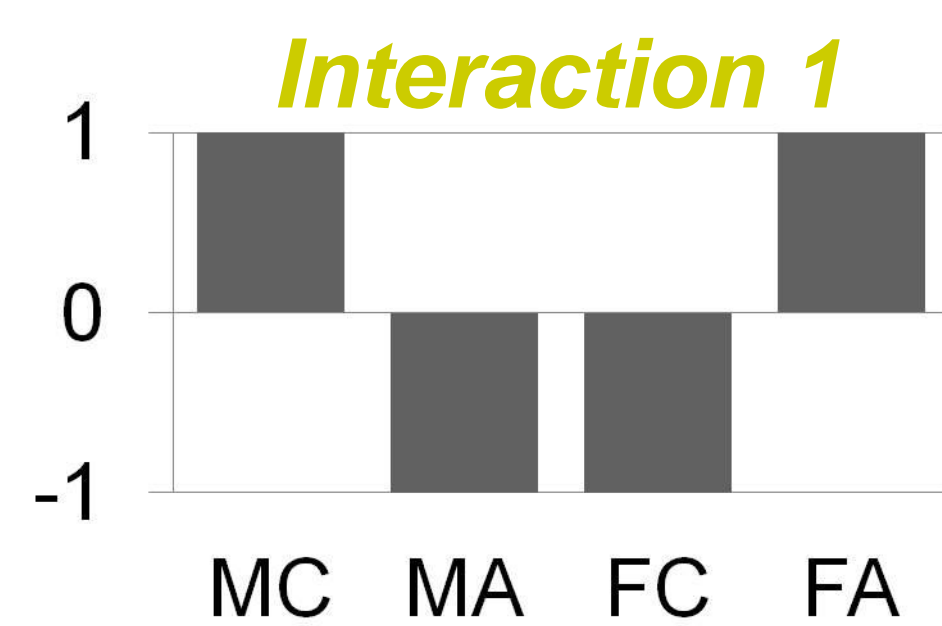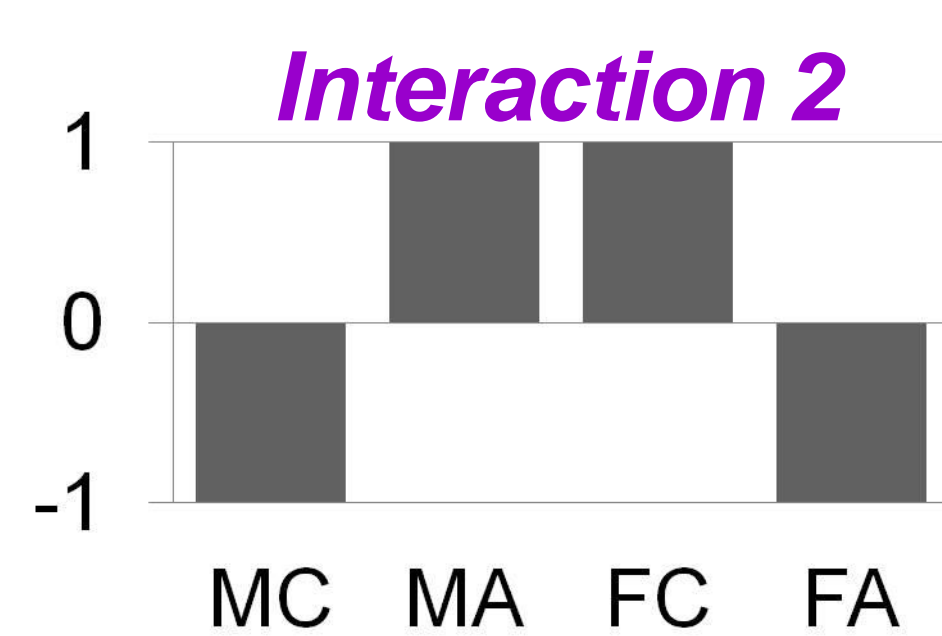

WM

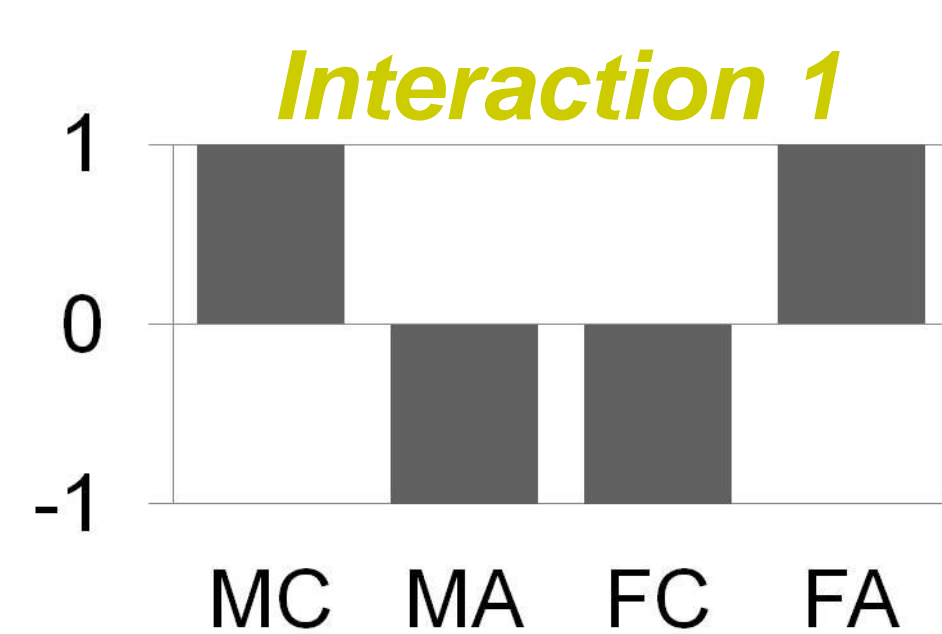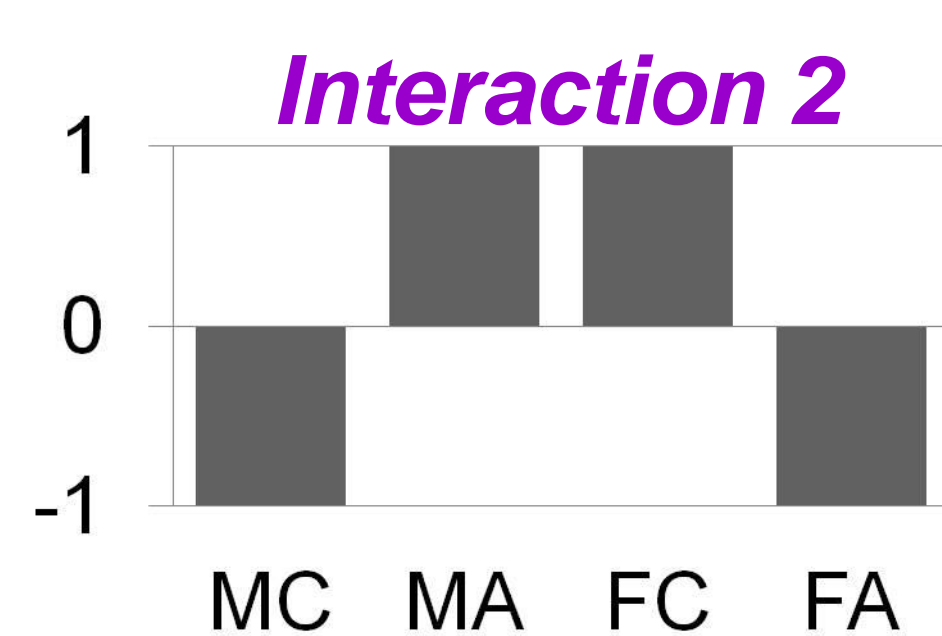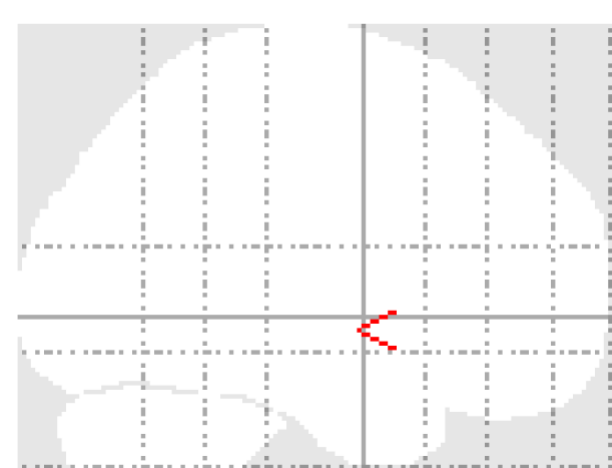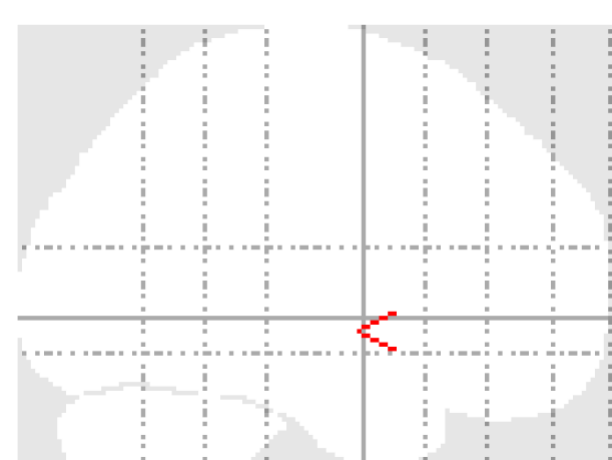

x = 25

x = 3

z = 12

x = 30

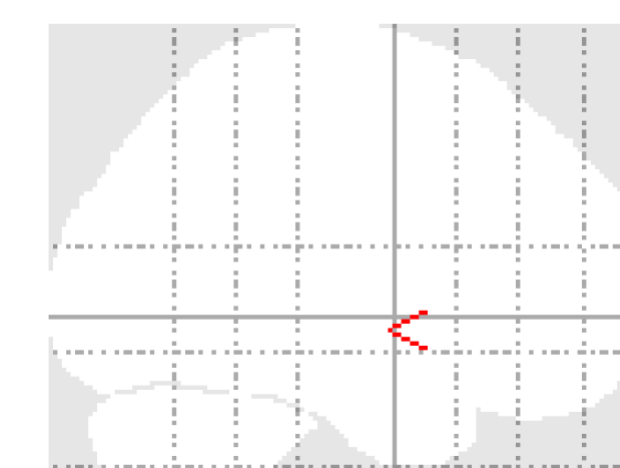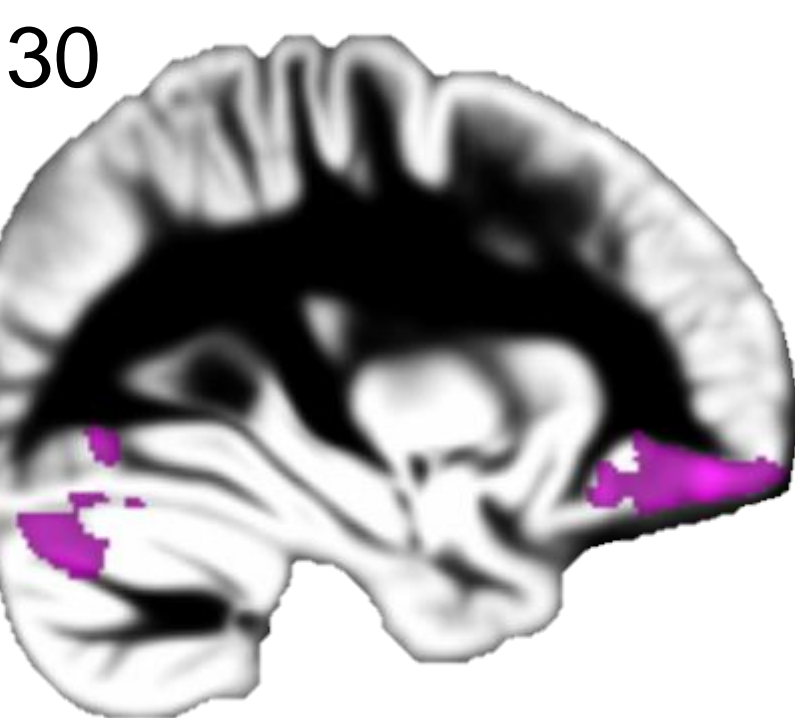

z = 30

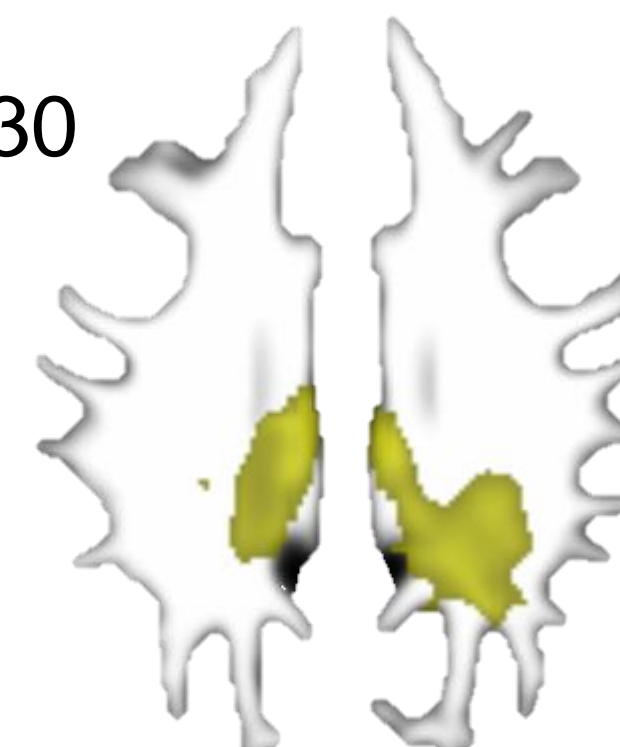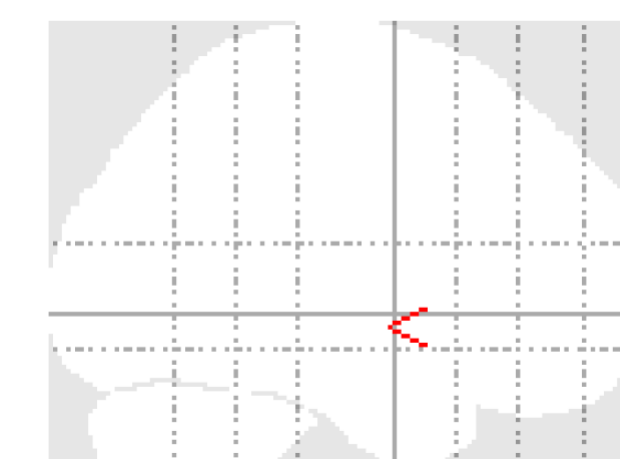

z = 30

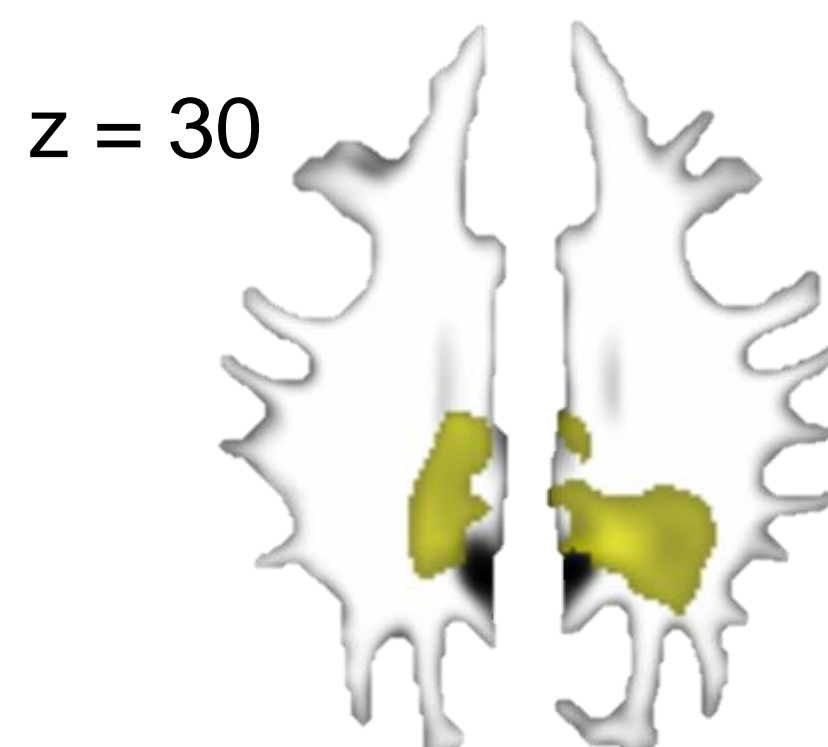

y = -19

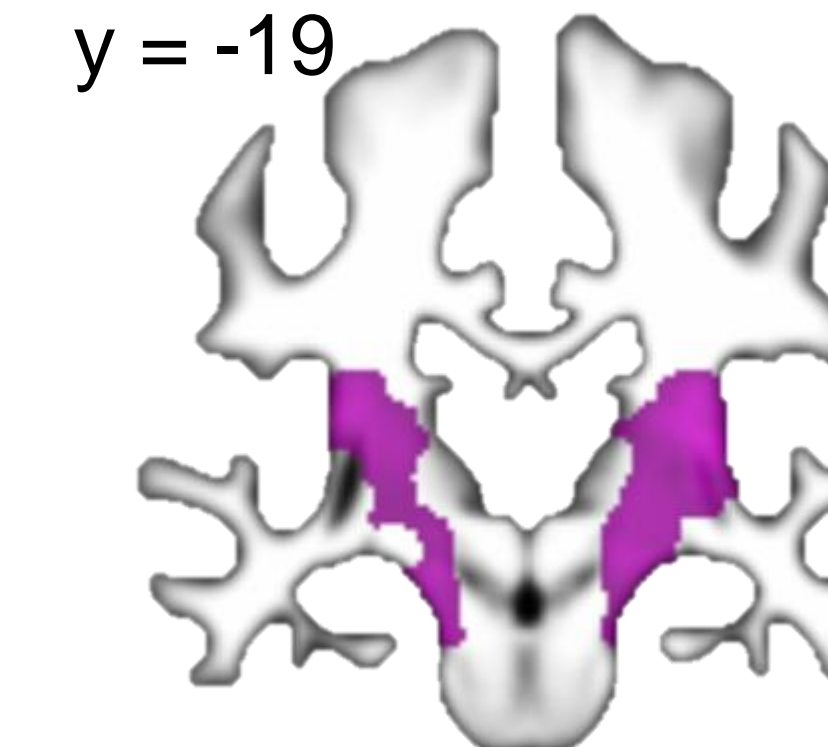

y = -19

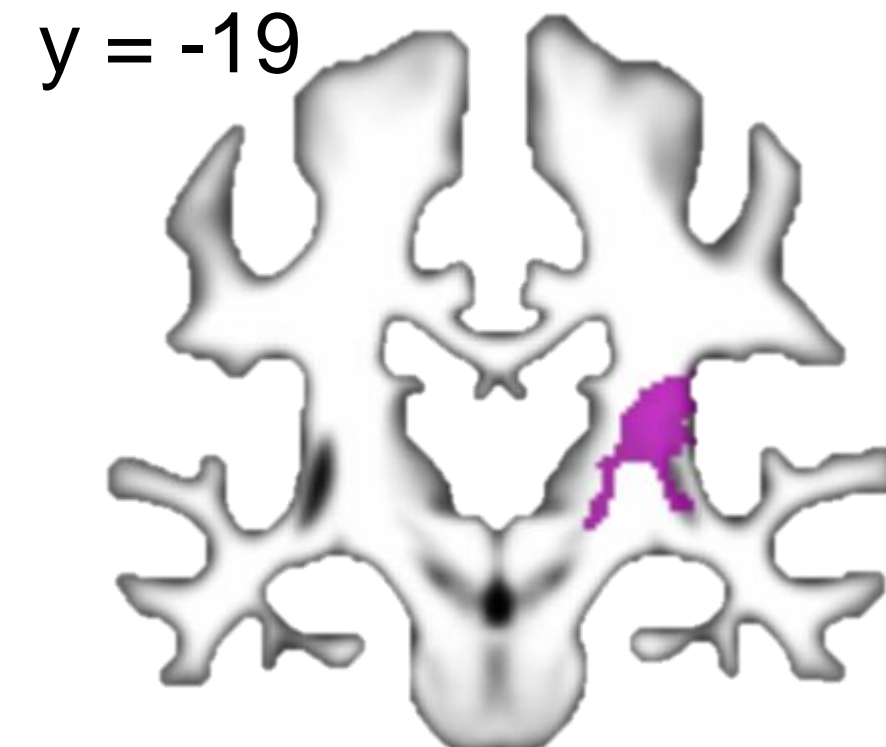

B

all

split1

split2

GM

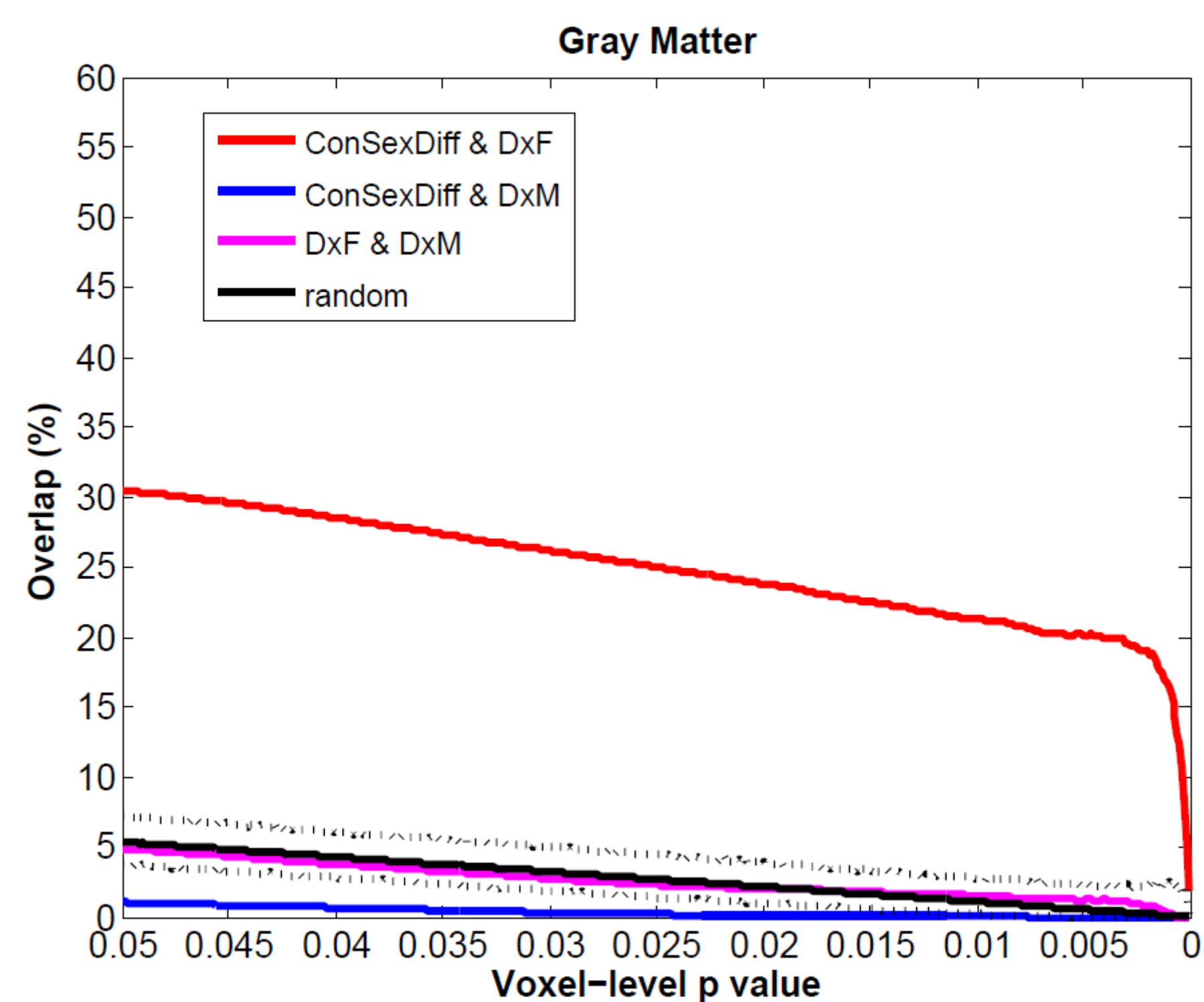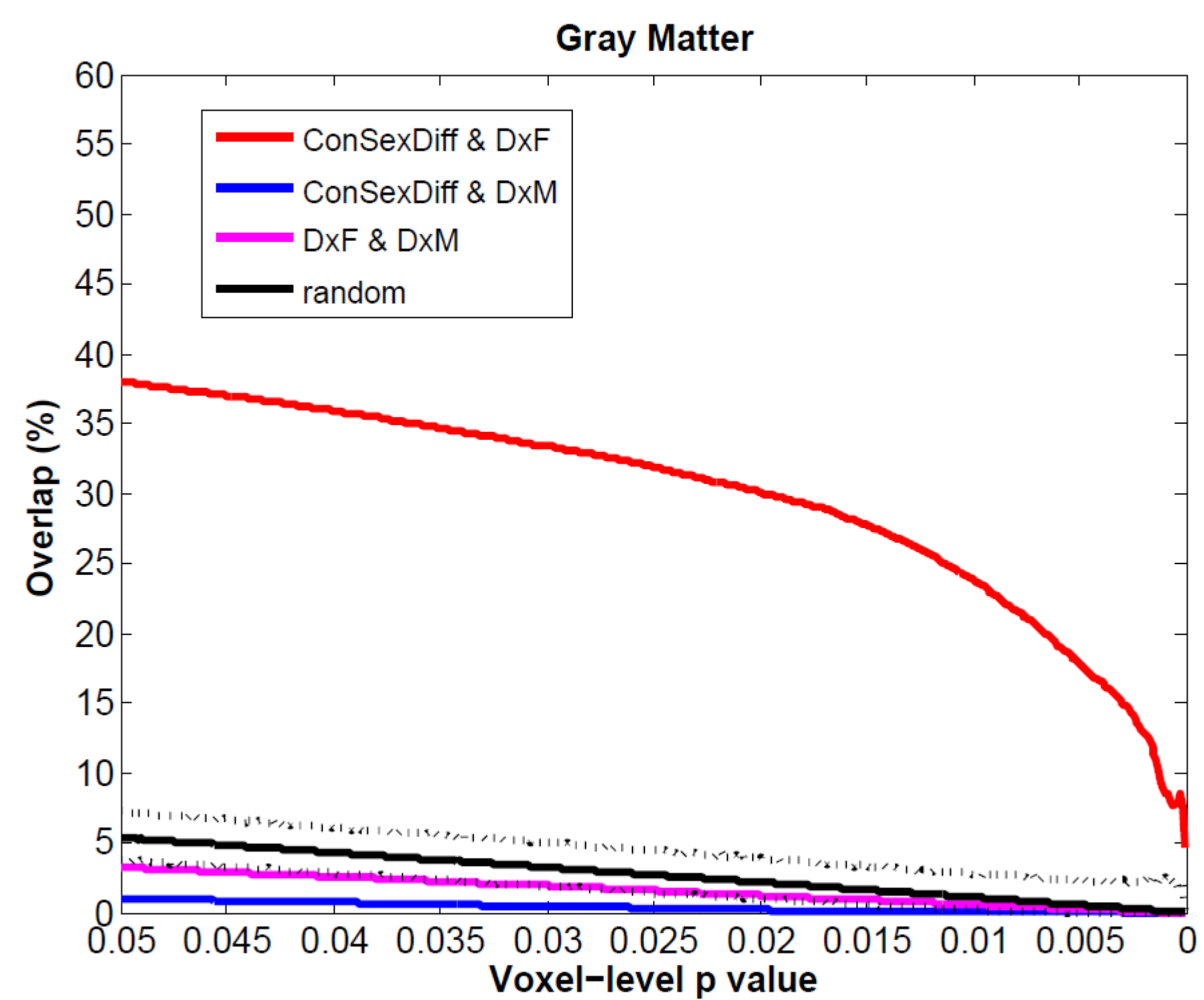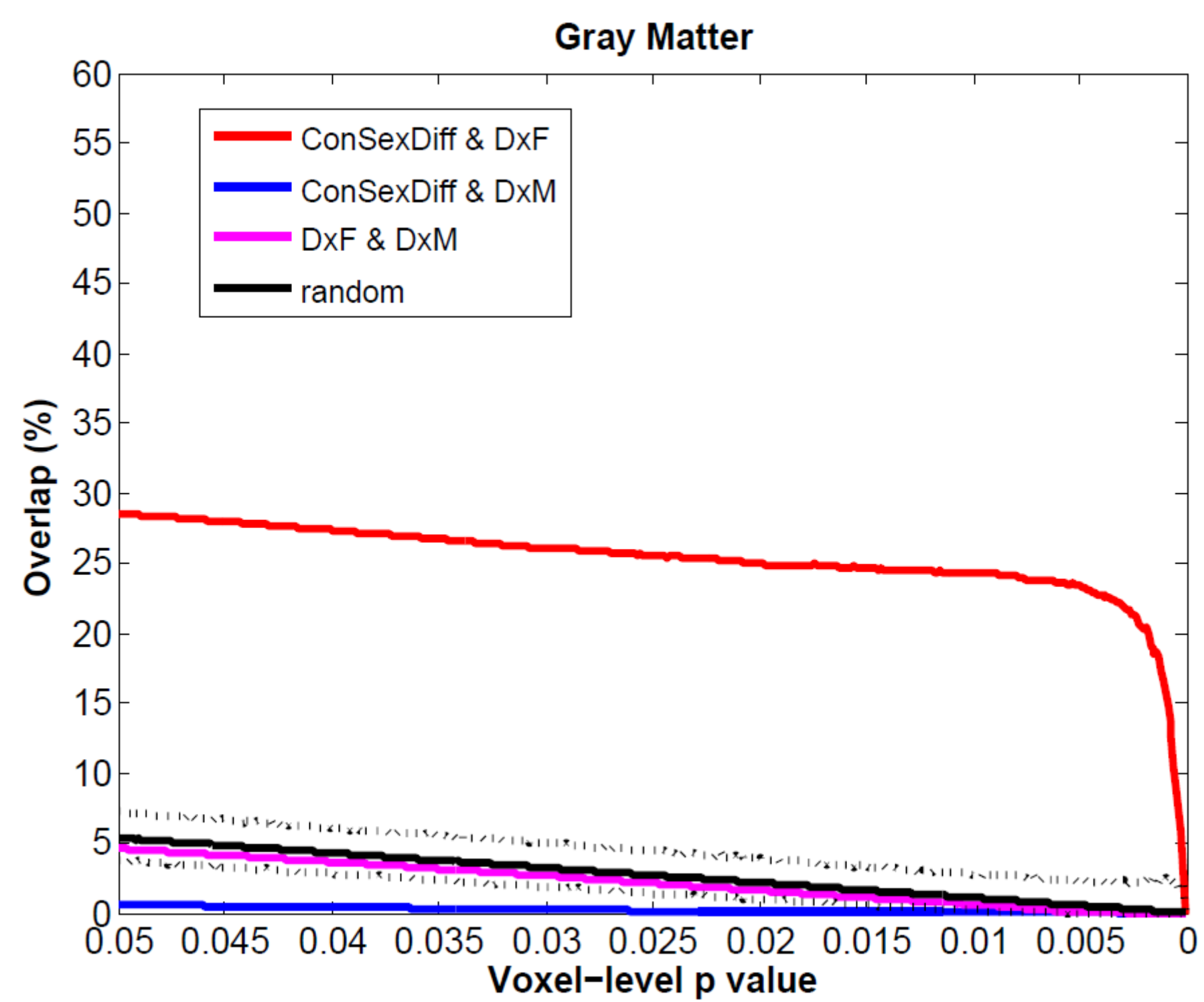

WM

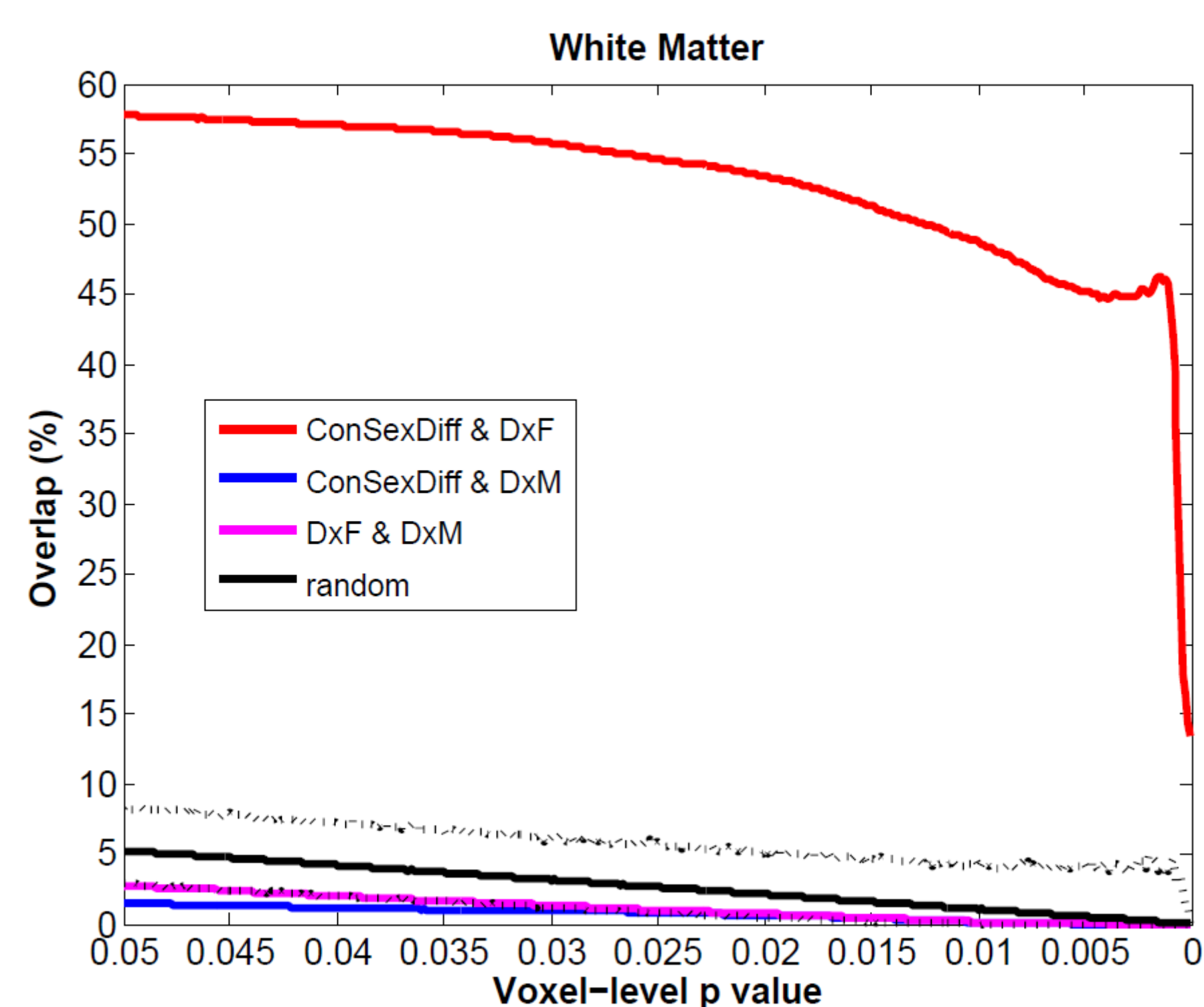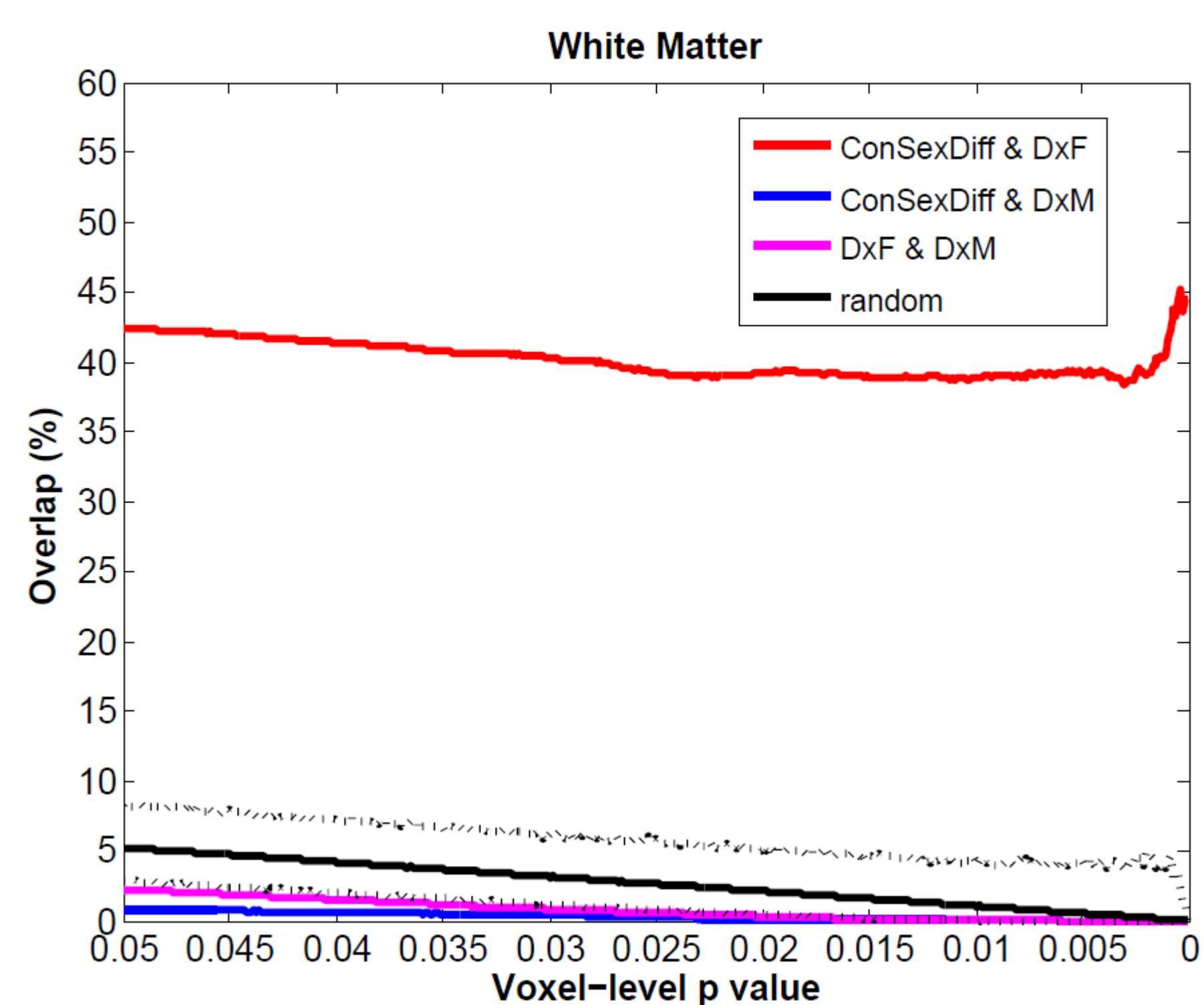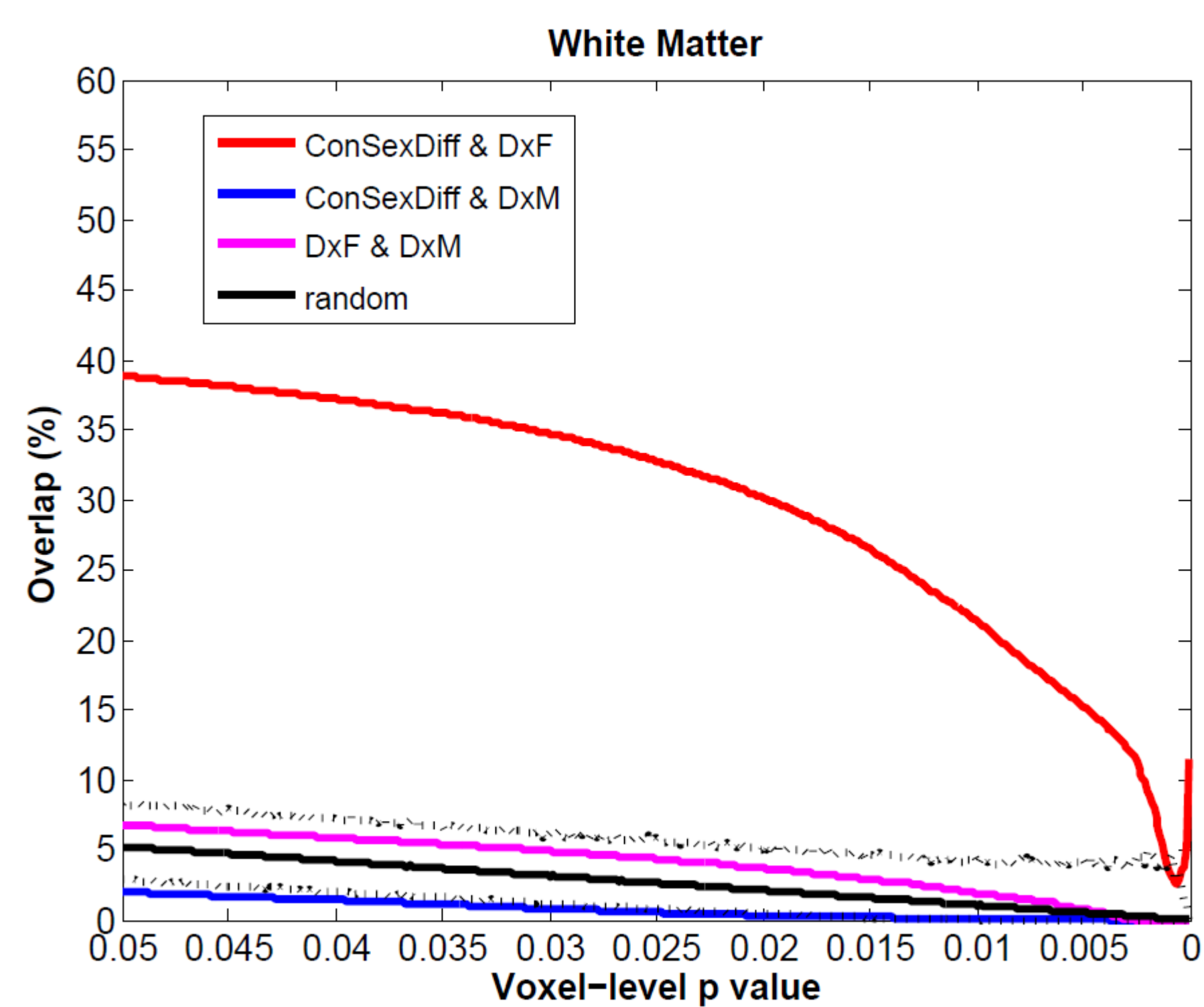

Supplement: Supplementary Data [file supp_awt216_brain-2013-00261-File012.pdf]
