# Supplementary material for: Biological sex affects the neurobiology of autism
Source: Brain. 2013 Aug 9;136(9):2799–815. doi: 10.1093/brain/awt216 (PMC3754459; doi:10.1093/brain/awt216)
Supplement: Supplementary Data [file supp_awt216_brain-2013-00261-File007.doc]

**Supplementary Material**

# Supplementary Methods

## Digit ratio measurement

This was only measured in females. Photocopies were taken using a high-resolution photocopier with participants’ palms pressed flat on the glass of the machine. Finger lengths of the second and fourth digits of both hands were measured from the midpoint of the basal crease to the tip of the finger with an electronic vernier calliper with an accuracy of 0.01 mm. This has previously been shown to be a reliable measure . One trained researcher measured all hands from photocopies twice. Intra-rater reliability across 105 participants, including the 60 female participants here, was excellent for all four digits (intra-class correlation coefficient > 0.99). The average of the two repeated measurements of each finger was taken as the final measure of finger length to calculate the 2D:4D ratio for the left and right hands.

## Structural MRI acquisition: Quantitative imaging

Driven Equilibrium Single Pulse Observation of T_1_ (DESPOT1) was utilized . In short, spoiled gradient recalled (SPGR) were acquired at two flip angles (α) from which an estimate of the absolute T_1_ value was derived at each voxel. These quantitative T_1_ maps were then used to create simulated structural T_1_-weighted inversion recovery (IR) images, with 176 contiguous slices (1mm x 1mm x 1mm resolution), a field-of-view of 25.6cm, a simulated repetition time/inversion time (TR/TI) of 1800/850ms, a scaling constant *ρ* = 10000 and a flip angle of 20°. This combination of parameters gave excellent deep and cortical gray/white matter contrast for tissue segmentation, without the need of modulation by B_0_ and B_1_ field inhomogeneities as compensation is introduced during the estimation of absolute values of T_1_. This quantitative imaging method has advantages over conventional qualitative T_1_-weighted imaging because it minimizes inter-scanner variance in MRI measurements and improves signal-to-noise contrast.

## A larger multi-center male sample additionally used for deriving MA–MC VBM comparison

An additional MA–MC VBM was conducted on a larger multi-center male sample (N=84/group) from the MRC AIMS project to provide greater power to detect the diagnostic group differences within males. This sample, collected from three sites (Institute of Psychiatry, King’s College London; Autism Research Centre, University of Cambridge; and Autism Research Group, University of Oxford), using a multi-center neuroimaging study platform, comprised 84 neurotypical adult males (London: 38, Cambridge: 31, Oxford: 15) and 84 males with autism (London: 38, Cambridge: 29, Oxford: 17) matched for age (MC: 28.0 yrs, SD = 6.3; MA: 26.1 yrs, SD = 7.1; *t* = 1.825, *p* = 0.070) and full-scale IQ (MC: 114.2, SD = 12.5; MA: 110.5, SD = 14.2; *t* = 1.784, *p* = 0.076) . Twenty-eight males with autism and 29 neurotypical males in the main sample of the present study were included in this larger sample; the slight difference in number of participants was due to group matching.

All participants were scanned using 3T MRI scanners fitted with an 8-channel receive–only RT head-coil: GE Medical Systems HDx, Department of Radiology, University of Cambridge; GE Medical Systems HDx, Centre for Neuroimaging Sciences, Institute of Psychiatry, Kings College London; Siemens Medical Systems Tim Trio, FMRIB Centre, University of Oxford. DESPOT1 was utilized to ensure standardization of structural MRI scans across the three scanner platforms. This well-validated protocol has been shown to have reliable and high gray/white matter contrast and has been extensively described elsewhere . As mentioned above, this quantitative imaging method has advantages in minimizing inter-scanner variance as well as improving signal-to-noise contrast. It has already been successfully implemented in multi-center/scanner studies to investigate gray and white matter variations associated with diagnostic status and individual differences in dispositional traits .

# Supplementary Results

## Behavioral features of the four groups

Compared to neurotypical females, females with autism reported significantly higher scores on the Autism Spectrum Quotient (AQ) (*t*_(58)_ = -16.9, *p* < 0.001), Toronto Alexithymia Scale (TAS-20) (*t*_(58)_ = -10.9, *p* < 0.001) and lower scores on the Empathy Quotient (EQ) (*t*_(58)_ = 15.4, *p* < 0.001), and performed less accurate on the ‘Reading the Mind in the Eyes’ Test (Eyes Test) (*F*_(1,56)_ = 16.0, *p* < 0.001, age and full-scale IQ as covariates). When all four groups were compared together in a 2x2 AN(C)OVA framework, there were significant main effects of diagnosis for all four measures (AQ: *F*_(1,116)_ = 319.5, *p* < 0.001; EQ: *F*_(1,116)_ = 250.8, *p* < 0.001; TAS-20: *F*_(1,116)_ = 164.7, *p* < 0.001; Eyes Test = *F*_(1,114)_ = 27.9, *p* < 0.001, age and full-scale IQ as covariates), along with a significant ordinal interaction for AQ (*F*_(1,116)_ = 12.4, *p* = 0.001) and EQ (*F*_(1,116)_ = 9.4, *p* = 0.003). There were no significant main effects of sex across all measures. Post-hoc comparisons between males and females with autism showed that they scored comparably on all measures except marginally higher AQ scores for females, corresponding to our previous reports . See Figure S1; also see main text Table 1 for descriptive statistics.

These indicate that overall adults with autism reported higher autistic traits, lower empathy, higher levels of alexithymia and had poorer mentalizing ability than neurotypical adults. Such effects were stronger in females than in males for AQ and EQ, to the extent of a statistically significant ordinal sex-by-diagnosis interaction. The patterns also showed that for cognitive features measured by the AQ, EQ and Eyes Test, both males and females with autism fitted predictions from the EMB theory , similar to earlier studies .

## How does brain morphology differ between adult females with and without autism?

Structural MRI images of the 30 females with and 30 without autism were processed according to the VBM pipeline described in the Methods section in the main text. All processing steps followed except that the study-specific template was generated only from these 60 female participants in DARTEL processing. Using the same thresholding method as applied in the 2x2 factorial design in the main text, statistical outcomes were corrected for multiple comparisons at the cluster level by controlling topological false discovery rate (FDR) calculated under Gaussian Random Field Theory , using a cluster-forming voxel-level height threshold of *p* < 0.025 for each contrast and a spatial extent threshold (corrected for non-stationarity ) that ensures a cluster-wise FDR at *q* < 0.05.

Globally, multivariate analysis of variance (MANOVA) with absolute (i.e., not adjusted by body size) total gray matter (GM), white matter (WM) and cerebrospinal fluid (CSF) volumes as dependent variables and diagnosis as fixed factor showed no significant main effect of diagnosis overall (Pillai’s Trace *V* = 0.062, *F*_(3,56)_ = 1.235, *p* = 0.306). Post-hoc ANOVA confirmed this in total GM (female controls, FC: mean = 824 cm^3^, standard deviation SD = 81 cm^3^; females with autism, FA: mean = 845 cm^3^, SD = 72 cm^3^; *F*_(1,58)_ = 1.190, *p* = 0.280), WM (FC: mean = 448 cm^3^, SD = 53 cm^3^; FA: mean = 465 cm^3^, SD = 47 cm^3^; *F*_(1,58)_ = 1.732, *p* = 0.193) and CSF (FC: mean = 236 cm^3^, SD = 53 cm^3^; FA: mean = 227 cm^3^, SD = 45 cm^3^; *F*_(1,58)_ = 0.548, *p* = 0.462) volumes.

At the local (voxel) level (Figure S2), VBM showed that females with autism had less relative GM volume (i.e., corrected for individual total) than neurotypical females in two clusters: one spread across bilateral subgenual, anterior and middle cingulate cortices (ACC/MCC), and supplementary motor areas (SMA) (cluster size k_e_ = 14,123 voxels, cluster-level FDR-corrected *q* < 0.001, peak-voxel MNI coordinate [12, 26, 27], *T* = 4.85); the other at cerebellar vermis lobules 6, 7, 8 and bilateral hemispheres lobule 6 (k_e_ = 5,254, cluster-level *q* = 0.001, peak-voxel [-4, -60, -28] *T* = 3.70). There were no significant clusters where females with autism had greater volumes than neurotypical females.

In WM, females with autism had smaller volumes in one cluster involving mainly the right ponto-cerebellar fibers (k_e_ = 8,033, cluster-level *q* = 0.001, peak-voxel [18, -21, -29] *T* = 3.86), and larger volumes in two bilateral clusters located near temporo-parieto-occipital cortices involving the arcuate fasciculus, cingulum, inferior longitudinal fasciculus, internal capsule, and the splenium of corpus callosum (right-lateralized cluster: k_e_ = 18,627, cluster-level *q* < 0.001, peak-voxel [34, -56, 18] *T* = 5.78; left-lateralized cluster: k_e_ = 17,305, cluster-level *q* < 0.001, peak-voxel [-16, -39, 26] *T* = 4.41).

The neuroanatomical features of autism in females identified here replicate observations from a previous smaller study that reveals no difference in global volume relative to neurotypical females, but smaller GM in ACC, smaller ponto-cerebellar fibers and larger bilateral temporo-parieto-occipital WM regions. This replication is noteworthy given they included independent adult samples different in age and IQ and were conducted with different acquisition and analysis protocols. This is in contrast to the frequent non-replication from male or male-predominant samples , suggesting that heterogeneity in the neural correlates of autism is reduced by examining females only.

## Split-half validation

To examine the replicability of the results to Questions 1 and 2, we performed a split-half validation. Each of the four groups was split in half by the order of subject recruitment, into odd-numbered (‘split1’) and even-numbered (‘split2’) subsamples (N=60, N=15/group). The same set of analyses was then performed on each subsample. See Figure S3.

For Question 1, both subsamples showed evidence for significant sex-by-diagnosis interactions in WM, largely identical in spatial location to that seen in the whole sample. However in each subsample, significant results were found in only one direction of contrast (which was different for the two subsamples), most likely caused by the decrease in statistical power due to halving the sample sizes. Similarly, in GM both subsamples showed significant sex-by-diagnosis interactions. However, the location of interaction effects occurred in distinct regions across each subsample, in the context of a marginally significant interaction in the whole sample.

Regarding Question 2, spatial overlap analyses in both subsamples showed comparable results and replicated observations seen within the whole sample. For both GM and WM, females, but not males, showed autism diagnosis effects that substantially and non-randomly (P < 0.001) overlapped with regions that were sexually dimorphic in neurotypical controls.

These results could be understood from the angle of heterogeneity in autism. The emergence of sex-by-diagnosis interactions in different brain regions in split-half subsamples suggests that subject heterogeneity may play an important role regarding *which* specific brain regions show sex-by-diagnosis interactions (though this seems to be less of an issue for WM since findings in split-half cross-validation are largely congruent with regions found in the whole sample, indicating that these specific regions may show relatively more consistent sex-by-diagnosis interactions). Different studies of similar size to these split-half subsamples may find different areas depending on which subgroup is sampled. Nevertheless, sex-by-diagnosis interaction, as a general theoretical concept, is commonly observed, suggesting its ubiquity in the neuroanatomy of autism.

On the other hand, the female-only EMB patterns in both GM and WM were replicated in the split-half subsamples despite reductions in statistical power due to halving the sample size. This may be where heterogeneity has less impact.

Regarding the role of sex-by-diagnosis interaction, an analogy from another domain of autism research might help: variability in the locus of mutations by copy number variations (CNVs) . CNVs are important for autism genetics, but there is currently a lack of specificity or consistency with respect to *where* such mutations will occur in any one individual (or a particular subgroup) with autism. By the same token, sex-by-diagnosis interaction may also be central to autism neuroanatomy, but *where* sex-specific neuroanatomy of autism occurs may vary as a function of subgroups. Without further stratification by subgroups, heterogeneity may remain the rule rather than the exception in studies looking for consistency in the anatomical location of abnormalities in autism. In spite of this, the split-half analyses are a proof of concept that such interactions are important across subgroups in autism.

# Supplementary Figure Legends

**Figure S1. Dispositional traits and mentalizing ability**

Line graphs (panels A, B, C, and D illustrate AQ, EQ, TAS-20, and Eyes Test respectively) indicate the interrelationship among the four groups under a 2x2 factorial AN(C)OVA framework. The squares indicate the estimated marginal mean for each group on the y-axis. The x-axis illustrates diagnostic groups with the neurotypical control groups on the left and autism groups on the right. The colors indicate sex with blue representing males and red representing females.

**Figure S2. Neuroanatomical features of adult females with autism**

A, Areas where females with autism have less relative GM volume compared to neurotypical females, overlaid on the GM segment of the study-specific template (from 60 females).

B & C, Areas where females with autism have less (orange clusters) or more (light-blue clusters) relative WM volume compared to neurotypical females, overlaid on WM tracts probabilistically defined from a human diffusion tensor imaging atlas . The right-sided figures repeatedly illustrate the same cluster involving different WM tract(s); purple: FWE p < 0.05 thresholded tracts; blue: percentage overlap amongst all participants (N=40) in the study creating this atlas > 50%; yellow: percentage overlap > 75%; red: percentage overlap > 90%. Coordinates are given in Montreal Neurological Institute (MNI) space.

Abbreviations: Cereb 6 = cerebellum hemisphere lobule 6, left and right; LACC = left anterior cingulate cortex; LsgACC = left subgenual ACC; LSMA = left supplementary motor area; RACC = right anterior cingulate cortex; RMCC = right middle cingulate cortex; RsgACC = right subgenual ACC; RSMA = right supplementary motor area; Ver 6-8 = cerebellum vermis lobules 6, 7 & 8 (Larsell’s schema).

**Figure S3. Split-half validation**

A, Results from the 2x2 factorial design analyses of GM and WM in the whole sample (column ‘all’) and the two split-half subsamples (columns ‘split1’ and ‘split2’). For GM, regions showing a significant sex-by-diagnosis interaction in the whole sample were marginal and thus represented by the empty ‘glass brain’ icon. However, in both split-half subsamples there were significant findings. In the split1 subsample sex-by-diagnosis interactions occurred in right cerebellum and occipital pole for one direction (‘Interaction 1’, yellow), and bilateral orbital medial prefrontal cortices, insula and superior temporal gyri for the other (‘Interaction 2’, dark purple). In the split2 subsample sex-by-diagnosis interactions occurred only for ‘Interaction 2’ direction, in right orbital lateral prefrontal cortex and cerebellum bilaterally. Interestingly, these regions showing significant interactions in the two subsamples do not overlap. For WM, regions showing significant interactions in the split1 subsample spatially replicated findings from the whole sample for ‘Interaction 2’ (although they were spatially less extensive). The same applied for the split2 subsample, that the findings were comparable in regions and direction (‘Interaction 1’) as those from the whole sample.

B, Spatial overlap analyses in both subsamples showed the same patterns as observed in the whole sample. This provides strong support for replicability.

# Supplementary References

Bagby RM, Parker JD, Taylor GJ. The twenty-item Toronto Alexithymia Scale--I. Item selection and cross-validation of the factor structure. J Psychosom Res. 1994 Jan;38(1):23-32.

Baron-Cohen S. The extreme male brain theory of autism. Trends Cogn Sci. 2002 Jun 1;6(6):248-54.

Baron-Cohen S, Wheelwright S. The empathy quotient: an investigation of adults with Asperger syndrome or high functioning autism, and normal sex differences. J Autism Dev Disord. 2004 Apr;34(2):163-75.

Baron-Cohen S, Wheelwright S, Hill J, Raste Y, Plumb I. The "Reading the Mind in the Eyes" Test revised version: a study with normal adults, and adults with Asperger syndrome or high-functioning autism. J Child Psychol Psychiatry. 2001 Feb;42(2):241-51.

Baron-Cohen S, Wheelwright S, Skinner R, Martin J, Clubley E. The autism-spectrum quotient (AQ): evidence from Asperger syndrome/high-functioning autism, males and females, scientists and mathematicians. J Autism Dev Disord. 2001 Feb;31(1):5-17.

Beacher FD, Minati L, Baron-Cohen S, Lombardo MV, Lai MC, Gray MA, et al. Autism attenuates sex differences in brain structure: a combined voxel-based morphometry and diffusion tensor imaging study. AJNR Am J Neuroradiol. 2012 Jan;33(1):83-9.

Chumbley JR, Friston KJ. False discovery rate revisited: FDR and topological inference using Gaussian random fields. Neuroimage. 2009 Jan 1;44(1):62-70.

Craig MC, Zaman SH, Daly EM, Cutter WJ, Robertson DM, Hallahan B, et al. Women with autistic-spectrum disorder: magnetic resonance imaging study of brain anatomy. Br J Psychiatry. 2007 Sep;191:224-8.

Deoni SC. High-resolution T1 mapping of the brain at 3T with driven equilibrium single pulse observation of T1 with high-speed incorporation of RF field inhomogeneities (DESPOT1-HIFI). J Magn Reson Imaging. 2007 Oct;26(4):1106-11.

Deoni SC, Williams SC, Jezzard P, Suckling J, Murphy DG, Jones DK. Standardized structural magnetic resonance imaging in multicentre studies using quantitative T1 and T2 imaging at 1.5 T. Neuroimage. 2008 Apr 1;40(2):662-71.

Devlin B, Scherer SW. Genetic architecture in autism spectrum disorder. Curr Opin Genet Dev. 2012 Jun;22(3):229-37.

Ecker C, Ginestet C, Feng Y, Johnston P, Lombardo MV, Lai MC, et al. Brain surface anatomy in adults with autism: the relationship between surface area, cortical thickness, and autistic symptoms. JAMA Psychiatry. 2013 Jan 1;70(1):59-70.

Ecker C, Suckling J, Deoni SC, Lombardo MV, Bullmore ET, Baron-Cohen S, et al. Brain anatomy and its relationship to behavior in adults with autism spectrum disorder: a multicenter magnetic resonance imaging study. Arch Gen Psychiatry. 2012 Feb;69(2):195-209.

Hayasaka S, Phan KL, Liberzon I, Worsley KJ, Nichols TE. Nonstationary cluster-size inference with random field and permutation methods. Neuroimage. 2004 Jun;22(2):676-87.

Lai MC, Lombardo MV, Chakrabarti B, Ecker C, Sadek SA, Wheelwright SJ, et al. Individual differences in brain structure underpin empathizing-systemizing cognitive styles in male adults. Neuroimage. 2012 Jul 16;61(4):1347-54.

Lai MC, Lombardo MV, Pasco G, Ruigrok AN, Wheelwright SJ, Sadek SA, et al. A behavioral comparison of male and female adults with high functioning autism spectrum conditions. PLoS ONE. 2011;6(6):e20835.

Lai MC, Lombardo MV, Ruigrok AN, Chakrabarti B, Wheelwright SJ, Auyeung B, et al. Cognition in males and females with autism: similarities and differences. PLoS ONE. 2012;7(10):e47198.

Manning JT, Scutt D, Wilson J, Lewis-Jones DI. The ratio of 2nd to 4th digit length: a predictor of sperm numbers and concentrations of testosterone, luteinizing hormone and oestrogen. Hum Reprod. 1998 Nov;13(11):3000-4.

Radua J, Via E, Catani M, Mataix-Cols D. Voxel-based meta-analysis of regional white-matter volume differences in autism spectrum disorder versus healthy controls. Psychol Med. 2011 Jul;41(7):1539-50.

Thiebaut de Schotten M, Ffytche DH, Bizzi A, Dell'Acqua F, Allin M, Walshe M, et al. Atlasing location, asymmetry and inter-subject variability of white matter tracts in the human brain with MR diffusion tractography. Neuroimage. 2011 Jan 1;54(1):49-59.

Via E, Radua J, Cardoner N, Happe F, Mataix-Cols D. Meta-analysis of gray matter abnormalities in autism spectrum disorder: should Asperger disorder be subsumed under a broader umbrella of autistic spectrum disorder? Arch Gen Psychiatry. 2011 Apr;68(4):409-18.
