# Supplementary material for: Biological sex affects the neurobiology of autism
Source: Brain. 2013 Aug 9;136(9):2799–815. doi: 10.1093/brain/awt216 (PMC3754459; doi:10.1093/brain/awt216)
Supplement: Supplementary Data [file supp_awt216_brain-2013-00261-File008.doc]

**Table S1. Clusters showing significant main and interaction effects in 2x2 factorial design VBM in gray matter**

| Region | Cluster size k_e_ (voxels) | Cluster-level *q* (FDR-corrected) | Peak-voxel  MNI coordinate (mm) | Peak-voxel T |
| --- | --- | --- | --- | --- |
| ***Male>Female*** |  |  |  |  |
| OPO, PCC, PCUN, Ling, Cal, TOJ, Sup Cblm, | 60253 | < 0.001 | 1, -96, 3 | 6.77 |
| FPO, DMPFC | 23672 | < 0.001 | 2, 55, 37 | 6.46 |
| Sensori-motor cortex | 3361 | 0.021 | 0, -11, 83 | 4.96 |
| STG, HG [left] | 5503 | 0.001 | -39, -35, 16 | 4.81 |
| Brainstem | 4267 | 0.006 | 5, -42, -65 | 4.61 |
| STG, HG [right] | 7477 | < 0.001 | 70, -17, 12 | 4.11 |
| ***Female>Male*** |  |  |  |  |
| OFC [right] | 4955 | 0.003 | 22, 24, -23 | 6.30 |
| THA, HIP, PHG, FG | 13802 | < 0.001 | 3, -12, 2 | 5.73 |
| Inf Cblm | 7290 | < 0.001 | -3, -75, -46 | 5.36 |
| SMA [left] | 3378 | 0.011 | -5, 9, 60 | 5.02 |
| OFC [left] | 4631 | 0.003 | -16, 31, -23 | 4.84 |
| SI [left] | 3923 | 0.007 | -23, -36, 65 | 4.80 |
| CAU [left] | 3301 | 0.011 | -13, 12, 10 | 4.70 |
| DLPFC [left] | 2909 | 0.018 | -44, 10, 37 | 4.70 |
| CAU [right] | 3380 | 0.011 | 14, 10, 10 | 4.61 |
| ***Autism>Control*** |  |  |  |  |
| MTG [left] | 4694 | 0.013 | -67, -24, -6 | 3.85 |
| ***Control>Autism*** |  |  |  |  |
| ACC, SMA | 5307 | 0.005 | -4, 36, 9 | 3.79 |

*Abbreviations*: ACC: anterior cingulate cortex; Cal: calcarine; CAU: caudate; DLPFC: dorsolateral prefrontal cortex; DMPFC: dorsomedial prefrontal cortex; FG: fusiform gyrus; FPO: frontal pole; HG: Heschl gyrus; HIP: hippocampus; Inf Cblm: inferior cerebellum; Ling: lingual gyrus; MTG: middle temporal gyrus; OFC: orbitofrontal cortex; OPO: occipital pole; PCC: posterior cingulate cortex; PCUN: precuneus; PHG: parahippocampal gyrus; SI: primary somatosensory cortex; SMA: supplementary motor area; STG: superior temporal gyrus; Sup Cblm: superior cerebellum; THA: thalamus; TOJ: temporo-occipital junction.
