# Supplementary material for: Biological sex affects the neurobiology of autism
Source: Brain. 2013 Aug 9;136(9):2799–815. doi: 10.1093/brain/awt216 (PMC3754459; doi:10.1093/brain/awt216)
Supplement: Supplementary Data [file supp_awt216_brain-2013-00261-File009.doc]

**Table S2. Clusters showing significant main and interaction effects in 2x2 factorial design VBM in white matter**

| Region | Cluster size k_e_ (voxels) | Cluster-level *q* (FDR-corrected) | Peak-voxel  MNI coordinate (mm) | Peak-voxel T |
| --- | --- | --- | --- | --- |
| ***Male>Female*** |  |  |  |  |
| OPO, ILF [left] | 7884 | < 0.001 | -13, -87, 10 | 5.81 |
| CC (Spln), AF [right] | 9665 | < 0.001 | 54, -35, 22 | 5.55 |
| CC (Spln), AF [left] | 3912 | 0.019 | -33, -29, 13 | 4.78 |
| FPO [right] | 7763 | < 0.001 | 12, 51, -8 | 4.78 |
| OPO, ILF [right] | 9419 | < 0.001 | 21, -72, 17 | 4.70 |
| FPO [left] | 8825 | < 0.001 | -15, 50, 14 | 4.43 |
| ***Female>Male*** |  |  |  |  |
| PCF, Brainstem | 20995 | < 0.001 | 8, -35, -49 | 6.31 |
| CC (Body), IC [left] | 14789 | < 0.001 | -22, -1, 36 | 4.98 |
| CC (Body), IC [right] | 6173 | 0.002 | 32, 13, 39 | 4.54 |
| ***Interaction 1***  ***(MA=MC, FA>FC)*** |  |  |  |  |
| Cing, ILF, CC (Spln), AF [right] | 11473 | < 0.001 | 35, -54, 16 | 4.38 |
| Cing, ILF, CC (Spln) [left] | 6409 | 0.002 | -18, -39, 25 | 3.86 |
| ***Interaction 2***  ***(MA>MC, FA<FC)*** |  |  |  |  |
| IC [left] | 5558 | 0.008 | -32, -9, 7 | 4.02 |
| IC [right] | 7234 | 0.003 | 32, -21, 7 | 3.56 |

*Abbreviations*: AF: arcuate fasciculus; CC (Body): body of corpus callosum; CC (Spln): splenium of corpus callosum; Cing: cingulum; FPO: frontal pole; IC: internal capsule; ILF: inferior longitudinal fasciculus; OPO: occipital pole; PCF: ponto-cerebellar fibers.
